# Ferroptosis Signature Shapes the Immune Profiles to Enhance the Response to Immune Checkpoint Inhibitors in Head and Neck Cancer

**DOI:** 10.1002/advs.202204514

**Published:** 2023-04-07

**Authors:** Chih‐Hung Chung, Chun‐Yu Lin, Chih‐Yu Chen, Chun‐Wei Hsueh, Yao‐Wen Chang, Chen‐Chi Wang, Pen‐Yuan Chu, Shyh‐Kuan Tai, Muh‐Hwa Yang

**Affiliations:** ^1^ Taiwan International Graduate Program in Molecular Medicine National Yang Ming Chiao Tung University and Academia Sinica Taipei 115201 Taiwan; ^2^ Institute of Clinical Medicine National Yang Ming Chiao Tung University Taipei 112304 Taiwan; ^3^ Department of Biological Science and Technology National Yang Ming Chiao Tung University Hsinchu 300093 Taiwan; ^4^ Institute of Bioinformatics and Systems Biology National Yang Ming Chiao Tung University Hsinchu 300093 Taiwan; ^5^ Institute of Data Science and Engineering National Yang Ming Chiao Tung University Hsinchu 300093 Taiwan; ^6^ Center for Intelligent Drug Systems and Smart Bio‐devices National Yang Ming Chiao Tung University Hsinchu 300093 Taiwan; ^7^ School of Dentistry Kaohsiung Medical University Kaohsiung 807378 Taiwan; ^8^ Cancer Progression Research Center National Yang Ming Chiao Tung University Taipei 112304 Taiwan; ^9^ Department of Biotechnology and Laboratory Science in Medicine National Yang Ming Chiao Tung University Taipei 112304 Taiwan; ^10^ School of Medicine National Yang Ming Chiao Tung University Taipei 112304 Taiwan; ^11^ Department of Otolaryngology Head & Neck Surgery Taichung Veterans General Hospital Taichung 40705 Taiwan; ^12^ Department of Post‐Baccalaureate Medicine College of Medicine National Chung Hsing University Taichung 40227 Taiwan; ^13^ Department of Otolaryngology Taipei Veterans General Hospital Taipei 112201 Taiwan; ^14^ Department of Oncology Taipei Veterans General Hospital Taipei 112201 Taiwan

**Keywords:** ferroptosis, head and neck cancer, programmed death ligand 1

## Abstract

As a type of immunogenic cell death, ferroptosis participates in the creation of immunoactive tumor microenvironments. However, knowledge of spatial location of tumor cells with ferroptosis signature in tumor environments and the role of ferroptotic stress in inducing the expression of immune‐related molecules in cancer cells is limited. Here the spatial association of the transcriptomic signatures is demonstrated for ferroptosis and inflammation/immune activation located in the invasive front of head and neck squamous cell carcinoma (HNSCC). The association between ferroptosis signature and inflammation/immune activation is more prominent in HPV‐negative HNSCC compared to HPV‐positive ones. Ferroptotic stress induces PD‐L1 expression through reactive oxygen species (ROS)‐elicited NF‐*κ*B signaling pathway and calcium influx. Priming murine HNSCC with the ferroptosis inducer sensitizes tumors to anti‐PD‐L1 antibody treatment. A positive correlation between the ferroptosis signature and the active immune cell profile is shown in the HNSCC samples. This study reveals a subgroup of ferroptotic HNSCC with immune‐active signatures and indicates the potential of priming HNSCC with ferroptosis inducers to increase the antitumor efficacy of immune checkpoint inhibitors.

## Graphical abstract

Ferroptosis stress elicits PD‐L1 expression in head and neck cancer cells and modulates tumor microenvironments to an immune‐active state, highlighting the potential of priming tumors with ferroptosis inducers to potentiate the antitumor efficacy of immunotherapy.

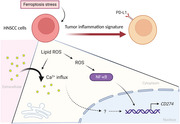

## 1 Introduction

Recent advances in understanding the new form of cell death, ferroptosis, which is different from other well‐known forms of cell death,^[^ ^1^ ^]^ have attracted the attention of cancer researchers due to the high potential of ferroptosis in cancer therapies. Ferroptosis is an iron‐dependent nonapoptotic cell death driven by the accumulation of lipid reactive oxygen species (ROS). Glutathione (GSH)/glutathione peroxidase 4 (GPX4) plays a pivotal role in protecting cells from ferroptosis by hydrolyzing lipid hydroperoxides.^[^ ^1^ ^]^ Mounting evidence supports the crucial role of ferroptosis in tumor suppression. For example, a strong dependence of cancer cells on GPX4 and system Xc‐ has been noted in multiple types of cancer,^[^ ^2^ ^]^ and the inhibition of the system Xc‐ coding gene *SLC7A11* induces ferroptotic cell death and enhances cytotoxicity in therapy‐resistant cancer cells.^[^ ^3^ ^]^ Importantly, cancer stem cells (CSCs) or mesenchymal‐like cancer cells have been found to be especially vulnerable to ferroptotic cell death.^[^ ^4^, ^5^ ^]^ This finding highlights the potential of ferroptosis induction as a new anticancer therapy, particularly targeting drug‐resistant CSCs.^[^ ^6^, ^7^ ^]^ However, the available strategy for ferroptosis induction as an adjunct to cancer therapy in clinical oncology is still limited.

In addition to the vulnerability of CSCs to ferroptotic cell death, another attracting point of ferroptosis in cancer treatment is that ferroptosis is closely related to the inflammatory response of the tumor microenvironment (TME). Ferroptosis impacts immune microenvironments in two different ways.^[^ ^8^ ^]^ First, ferroptosis affects the number and function of immune cells themselves. Ferroptosis influences macrophage polarization to modulate immune environments.^[^ ^9^ ^]^ Lipid peroxidation and ferroptosis regulate the viability and activity of CD8^+^ cytotoxic T cells and CD4^+^ helper T cells.^[^ ^10^, ^11^ ^]^ Second, ferroptotic cells themselves release damage‐associated molecular patterns (DAMP) which can be recognized by immune cells to trigger subsequent inflammatory responses.^[^ ^12^, ^13^ ^]^ Ferroptotic cells also produce abundant pro‐inflammatory cytokines and chemokines to create an inflamed microenvironment.^[^ ^14^, ^15^ ^]^ However, the clinical relevance and therapeutic impact of the signature created by endogenous ferroptotic stress in tumor cells, the “ferroptotic signature,” remains elusive.

The clinical characteristics of head and neck squamous cell carcinoma (HNSCC) are distinct from those of cancers originating from other tissues/organs: advanced HNSCC is often associated with severe destruction of surrounding tissues and neck lymphadenopathy, and local‐regional recurrence is the main pattern of treatment failure.^[^ ^16^ ^]^ In recent years, the great success of anticancer immunotherapy, especially immune checkpoint inhibitors (ICIs), has led to a paradigm shift in cancer treatments.^[^ ^17^ ^]^ In HNSCC, ICIs showed promising results in recurrent/metastatic (R/M) HNSCC.^[^ ^18^, ^19^, ^20^ ^]^ The programmed death ligand‐1 (PD‐L1) is expressed on the surface of multiple types of cells in TME including cancer cells and is considered a major indicator for ICI treatments.^[^ ^21^, ^22^ ^]^ However, clinical strategies to enhance PD‐L1 expression and remodel the immune environment to increase ICI efficacy are limited. Identifying the ICI‐susceptible subgroup of HNSCC and developing a strategy to sensitize HNSCC to ICI treatment are urgent and unmet medical needs.

In this study, we demonstrate that ferroptotic stress induces the inflammation signature and PD‐L1 expression in HNSCC. We also reveal that the ferroptosis signature of HNSCC shapes TME to an active immune state. This finding provides a potential strategy to enhance the efficacy of ICIs by identifying the optimal group of patients for treatment and priming HNSCC with potential ferroptosis inducers.

## 2 Results

### 2.1 The Ferroptosis Signature Correlates with Inflammation in HNSCC Samples

**Figure 1:**
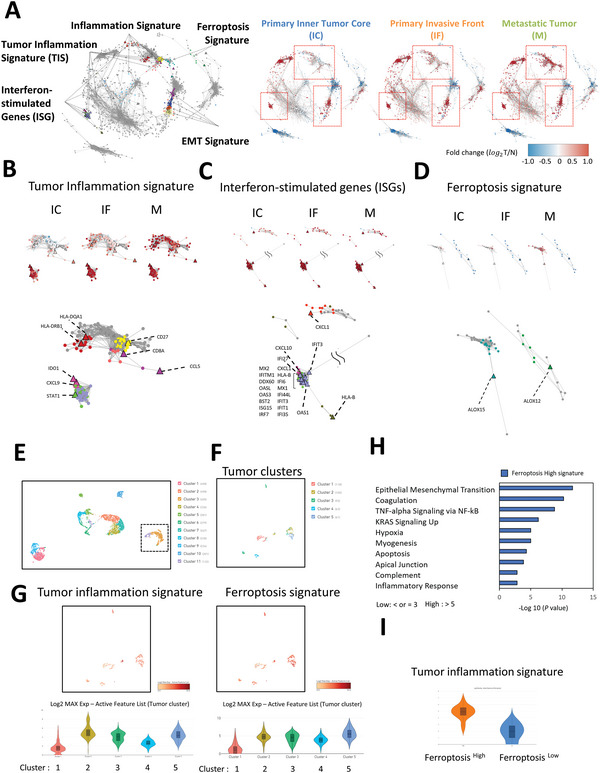
Correlation between ferroptotic and inflammation‐related signature in HNSCC. A) Gene correlation network constructed by weighted gene coexpression network analysis (WGCNA), including 1447 nodes and 12 037 edges. The nodes are colored by the corresponding module membership, and details of the modules with representative enriched biological terms are provided in Figure S1B, Supporting Information. The enriched ferroptosis, inflammation, tumor inflammation, EMT, interferon‐stimulated gene signatures are shown for distinct modules. Right, network with overlay of the log_2_T/N values for inner tumor core (IC), invasive front (IF), and metastatic tumor (M) in HNSCC samples. B) Subnetworks for the tumor inflammation signature in the IC, IF, and M. Corresponding genes (triangle) for the tumor inflammation signature are shown. C) Subnetworks for the interferon‐stimulated gene (ISG) signature in IC, IF, and M. Corresponding genes (triangle) for the tumor inflammation signature are shown. D) Subnetworks for the ferroptosis signature in the IC, IF, and M. Corresponding genes (triangle) for the ferroptosis signature are shown. E) A UMAP plot of a total of 3369 cells generated 11 clusters from the primary tumors of three HNSCC patients. The cell counts for each cluster are indicated in brackets. The tumor cell clusters determined by the epithelial score are indicated in squares. F) A UMAP plot of tumor cells re‐clustered in 5 groups. G) UMAP (upper) and violin plots (lower) showing the expression of tumor inflammation signature (left) and ferroptosis (right) signatures in 5 tumor cell clusters. H) Gene ontology analysis of the signal pathways in the ferroptosis_high group of HNSCC scRNA‐seq samples. The high and low groups were defined by the ferroptosis signature log2 expression > 5 as the high group and ≤3 as low group. I) Violin plots for showing the expression of tumor inflammation signature in ferroptosis high and low group.

To systemically approach the heterogeneous characteristics of HNSCC representing different steps in the metastatic journey, we collected matched pairs of primary tumors (including the inner core of the tumor, IC; and the invasive front, IF), metastatic tumors (M), and adjacent normal tissues from 21 patients with HNSCC receiving treatments at Taipei Veterans General Hospital (TVGH). Bulk RNA sequencing (RNA‐seq) was performed from the 65 collected samples (Figure S1A, Table S1, Supporting Information). Weighted correlation network analysis (WGCNA) was implemented for 18428 protein‐encoding genes from the samples^[^ ^23^ ^]^ (Figure S1B, Table S2, Supporting Information). Differential patterns of signaling networks, especially inflammation/immune‐related signatures and ferroptosis signals, were observed among the three parts of the tumors. The epithelial‐mesenchymal transition (EMT) signature was also observed to validate the spatial gene expression relationship in the collected samples (**Figure** **1**A). Therefore, we annotated the modules with a hypergeometric test in the ferroptosis signature (with reference to WP_FERROPTOSIS),^[^ ^24^ ^]^ microenvironmental inflammation (with reference to BIOCARTA_INFLAM_PATHWAY), tumor inflammation,^[^ ^25^ ^]^ EMT,^[^ ^26^ ^]^ and interferon‐stimulated gene (ISG)^[^ ^27^ ^]^ signature sets (Table S3, Supporting Information). For EMT, an enriched signature from IC to IF to M was observed by network analysis with overlay of the log_2_T/N values between tumors (T) and corresponding normal tissues (N) (Figure S1C,E, Supporting Information), which validated the spatial gene expression pattern of the samples. Regarding the subnetworks centered on the ferroptosis and immune‐related signature modules, a gradual increase in the expression of microenvironmental inflammation signature (Figure S1D,E, Supporting Information), tumor inflammation (Figure 1B; Figure S1E, Supporting Information), ISG (Figure 1C; Figure S1E, Supporting Information), and ferroptosis (Figure 1D; Figure S1E, Supporting Information) from IC to IF to M was revealed. Data indicate that tumor aggressiveness was associated with EMT and inflammation as expected, and an increased ferroptosis signature was also revealed together with increased aggressiveness.

Next, we applied single‐cell RNA sequencing (scRNA‐seq) to three primary HNSCC samples to investigate the correlation between tumor inflammation and ferroptosis. The characteristics of the patient are shown in Figure S1A and Table S1, Supporting Information. The unsupervised clustering of 3369 cells generated 11 clusters of cells (Figure 1E). We identified tumor cells or non‐tumor cells population from scRNA seq data according to an epithelial score^[^ ^28^ ^]^ (Figure S1F, Supporting Information). We analyzed the signature of microenvironment cells, including T cells, B/plasma cells, dendritic cells, macrophages, fibroblast, myocytes mast cells, and endothelial cells (Figure S1G, Supporting Information). Next, we focused on the analysis of tumor cell clusters (comprising clusters 3 and 11 in the dotted square box of Figure 1E). A re‐clustering of tumor cells generated five clusters distributed by UMAP (Figure 1F; Table S4, Supporting Information). The correlation of tumor inflammation and ferroptosis signature was demonstrated between tumor cell groups (Figure 1G; Table S5, Supporting Information). MSigDB hallmark analysis was used to compare the expression of the top 50 genes identified by scRNA‐seq in ferroptosis_high versus ferroptosis_low cells. The results showed that the EMT and inflammation response were significantly enriched signatures in ferroptosis_high cells (Figure 1H). We also found that ferroptosis_high cells expressed a higher tumor inflammation signature compared to ferroptosis_low cells (Figure 1I). Taken together, these results indicate a significant association of ferroptosis signature and inflammation in HNSCC samples.

### 2.2 Spatial Association between the Ferroptosis Signature and Tumor Inflammation in HNSCC

**Figure 2:**
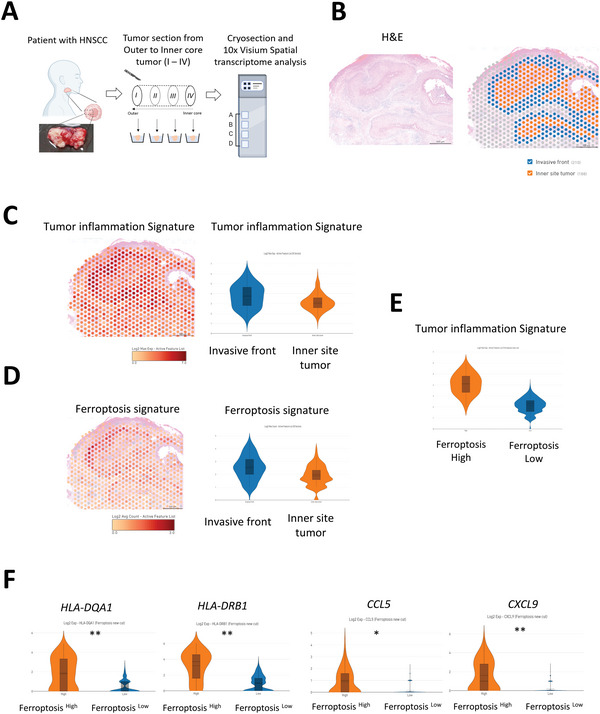
Spatial correlation between ferroptotic and inflammation‐related signature in HNSCC. A) A schema to show the workflow of Visium spatial transcriptomic analysis of a HNSCC tumor sample. B) H&E staining of the HNSCC tumor sample and the tumor invasive fronts region and the inner tumor region in one of the section tumors. C) Localization of the expression of the tumor inflammation signature in the tissue (left). Violin plots to show the expression of tumor inflammation signature in the invasive front and the inner core of the tumor (right). D) Localization of the expression of the ferroptosis signature in the tissue (left). Violin plots to show the expression of ferroptosis signature in the invasive front and the inner tumor core (right). E) Violin plots to show the expression of the tumor inflammation signature in ferroptosis high and low group in Visium analysis. F) Violin plots to show the inflammation‐related genes (*HLA‐DQA1*, *HLA‐DRB1*, *CCL5*, and *CXCL9)* which were generated by software 10× Loupe Browser 6.2 and adjusted using the Benjamin–Hochberg correction for multiple tests.

To track the ferroptosis signature throughout the metastatic journey of HNSCC cells, we applied the 10x Genomics Visium spatial transcriptomic platform for analyzing two independent HNSCC samples. The characteristics of the patients are shown in Table S1, Supporting Information. The workflow of 10x Genomics Visium spatial transcriptomic analysis is illustrated in **Figure** **2**A. The samples were harvested immediately after surgery and then divided the samples from the outer part of the tumor to the inner tumor part by cryosection and analyzed using 10x Genomics Visium. For patient no. 1, unsupervised clustering generated 12 clusters in four analyzed slides (Figure S2A, Supporting Information). The distribution of the tumor and non‐tumor clusters corresponded to the tumor and stromal parts in H&E staining (Figure S2B, Supporting Information). We also analyzed the gene expression of microenvironmental cells in the tested samples (Figure S2C,D, Supporting Information).

Here, we identified the tumor invasive fronts region and the inner tumor region in one of the section tumors (Figure 2B). A similar spatial distribution pattern of tumor inflammation and ferroptosis signatures was observed in the samples analyzed by 10x Genomics Visium (Figure 2C,D). Next, we focused on tumor cell clusters (clusters T1–T6). The ferroptosis_high group revealed a higher expression level of the tumor inflammation signature (Figure 2E). An increased expression of the inflammation/immune‐related genes *HLA‐DQA1*, *HLA‐DRB1*, *CCL5*, and *CXCL9* was shown in the ferroptosis_high group (Figure 2F). The positive correlation between tumor inflammation and ferroptosis signatures was validated in patient no. 2 (Figure S3, Supporting Information). Together, spatial transcriptomic studies further support the colocalization of tumor inflammation and ferroptosis signatures in the same histological regions of HNSCC.

### 2.3 Ferroptosis Induces the Immunogenic Signature and PD‐L1 Expression in HNSCC

**Figure 3:**
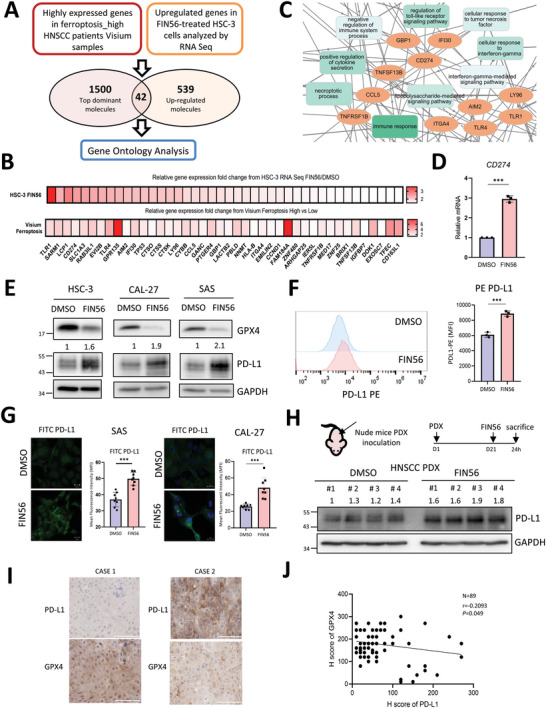
Ferroptosis inducers upregulate PD‐L1 expression. A) A flowchart to illustrate the strategy of identifying the major genes influenced by ferroptosis induction in HNSCC. The top 1500 genes expressed in the high ferroptosis signature of the Visium analysis and 539 upregulated genes (FPKM fold change ≥ 1.5) in FIN56‐treated HNSCC HSC‐3 cells compared to the control. B) A heatmap to show the expression of 42 co‐upregulated genes in panel (A) by relative fold change of FPKM value in HSC‐3 RNA‐seq (upper) and Visium (lower) analyses. C) A zoom‐in picture of the GO ontology analysis for identifying the hub gene of HNSCC under ferroptosis stress. D) RT‐qPCR to examine the relative mRNA level of *CD274* in HSC‐3 cells treated with FIN56 1 µm for 48 h or vehicle control. The data is presented in mean ± S.D, *n* = 3. ***p < 0.001 (Student's *t*‐test). E) Western blots to examine the expression of GPX4 and PD‐L1 in HNSCC cell lines HSC‐3, CAL‐27, and SAS cells treated with FIN56 1 µm for 48 h or a vehicle control. GAPDH was a loading control. F) Flow cytometry for detecting the PD‐L1 expression of HSC‐3 cells treated with FIN56 1 µm for 48 h or a control vehicle DMSO. The data is presented in mean ± S.D, *n* = 3. ***p < 0.001 (Student's *t*‐test). G) Immunofluorescent imaging to detect the membranous FITC‐PD‐L1 signal in SAS (left) and CAL‐27 (right) cells treated with FIN56 1 µm for 24 h versus a vehicle. Scale bar = 20 µm. Quantification of FITC intensity by imageJ. The data is presented in mean ± S.D, *n* = 8. ***p < 0.001 (Student's *t*‐test). H) Up, a schema for patient‐derived xenograft (PDX) tumors. HNSCC PDX tumors were inoculated into the subcutaneous region of nude mice. FIN56 (100 mg kg^−1^) were intratumorally injected 21 days after tumor inoculation. The mice were sacrificed after 24 h. The tumors were harvested for experiments. Down, western blot of PD‐L1 of the xenografted tumors treated with FIN56 or a vehicle control. GAPDH was a loading control. I) Representative results of immunohistochemical staining for PD‐L1 and GPX4 in tumor slides from HNSCC patients. Scale bar = 100 µm. J) Correlation between the expression of *GPX4* and *CD274* in immunohistochemical staining of PD‐L1 and GPX4 (*n* = 89) from the HNSCC tissue array. Pearson's correlation coefficient and the corresponding *p*‐value are shown.

Our observation of the clinical sample suggested that the tumor inflammation signature was correlated with the ferroptosis signature of HNSCC (Figures 1, 2). Therefore, we sought the group of major genes influenced by ferroptotic stress in HNSCC. To examine the impact of non‐lethal ferroptotic stress on HNSCC cells, we applied two ferroptosis inducers, (1S,3R)‐RSL3 (RSL3) and FIN56^[^ ^29^ ^]^ to treat HNSCC. The mechanism of action of these two drugs is summarized in Figure S4A, Supporting Information. A panel of HNSCC cell lines and a primary HNSCC culture with different spectrums of EMT phenotype (Figure S4B, Supporting Information) were subjected to the lipid ROS analysis and the WST‐1 assay under treatment with ferroptosis inducers. Epithelial‐type FaDu and SAS cells were more resistant to the ferroptosis inducers RSL3 and FIN56 and generated fewer lipid ROS after treatment, and mesenchymal‐type OECM‐1 cells were the most susceptible to inhibition of GPX4 among these cell lines and generated the most lipid ROS after treatment (Figure S4C–E, Supporting Information). We applied sublethal doses of ferroptosis inducers to treat HNSCC cells and examined the ferroptotic stress‐induced transcriptomic change in subsequent experiments (Figure S4F, Supporting Information). The experimental schema is illustrated in **Figure** **3**A. We intersected the genes up‐regulated in HNSCC samples analyzed by two platforms: the top 1500 genes up‐regulated in ferroptosis_high group by spatial transcriptomics (Table S6A, Supporting Information) and the genes up‐regulated in the RNA sequencing results of HSC‐3 cells treated with a sublethal concentration of FIN56 versus control (*n* = 539) (FPKM fold change ≥ 1.5) (Table S6B, Supporting Information). 42 genes were found to be co‐upregulated in both platforms (Figure 3B; Table S6C, Supporting Information), and these genes were subjected to GO ontology analysis. Interestingly, *CD274* acts as the signal hub in these upregulated genes (Figure S5A, Supporting Information and a zoomed‐in illustration in Figure 3C; Table S7, Supporting Information), and upregulation was validated by RT‐qPCR in HSC‐3 cells treated with FIN56 (Figure 3D). In subsequent experiments, we focused on ferroptosis‐induced *CD274* due to the high therapeutic impact of up‐regulation of PD‐L1 in cancer cells. Treatment of HNSCC cells (HSC‐3, CAL‐27, and SAS) with a sublethal dose of FIN56 upregulated the expression of the PD‐L1 protein (Figure 3E). The increase in membranous PD‐L1 expression was demonstrated in HNSCC cells under FIN56 treatment by flow cytometry (Figure 3F) and immunofluorescent staining (Figure 3G). FIN56 treatment enhanced PD‐L1 expression in the post‐treatment samples of patient‐derived xenografts (Figure 3H). The ferroptosis inhibitor ferrostatin‐1 reversed the up‐regulation of PD‐L1 induced by RSL3 in HSC‐3 cells (Figure S5B, Supporting Information). An analysis of TCGA HNSCC data showed that *CD274* expression was negatively correlated with the level of ferroptosis inhibition genes (*GPX4*, *GSS*, *GCLC*, *SLCA11*, *SLC3A2*) and positively correlated with the ferroptosis promotion gene *ACSL4* (Figure S5C,D, Supporting Information). A decrease in the expression of the ferroptosis inhibition genes (*GPX4*, *GSS*, *GCLC*, *SLCA11*, *SLC3A2*) and an increase in *ACLS4* was observed in the *CD274*_high group (Figure S5E, Supporting Information).

Next, we performed a subgroup analysis to explore the correlation between the expression of *CD274* and ferroptosis‐related genes in HPV‐positive versus negative HNSCC because HPV positivity represents distinct clinical characteristics of HNSCC patients. A prominent correlation was shown between the expression of *CD274* and ferroptosis‐related genes in the HPV‐negative group, that is, a negative correlation of *CD274* and ferroptosis‐related genes (*GPX4*, *GSS*, *GCLC*, *SLC3A2*) and a positive correlation of *CD274* and *ACSL4* (Figure S6A, Supporting Information). However, the correlation between *CD274* and ferroptosis‐related genes was not as evident in HPV‐positive cases, with the exception of the positive correlation between *CD274* and *ACSL4* (Figure S6B, Supporting Information). Furthermore, we examined the expression level of the PD‐L1 and GPX4 proteins in the TVGH HNSCC tumor samples, and a significant negative correlation between PD‐L1 and GPX4 was revealed (Figure 3I,J). The above data indicate a negative correlation between the expression of PD‐L1 and ferroptosis‐related genes in HNSCC samples, especially those negative for HPV, and a sublethal stress of ferroptosis up‐regulates PD‐L1 in HNSCC cells.

### 2.4 Ferroptosis‐Induced NF‐*κ*B Activation and Calcium Influx Contribute to Up‐Regulation of PD‐L1

**Figure 4:**
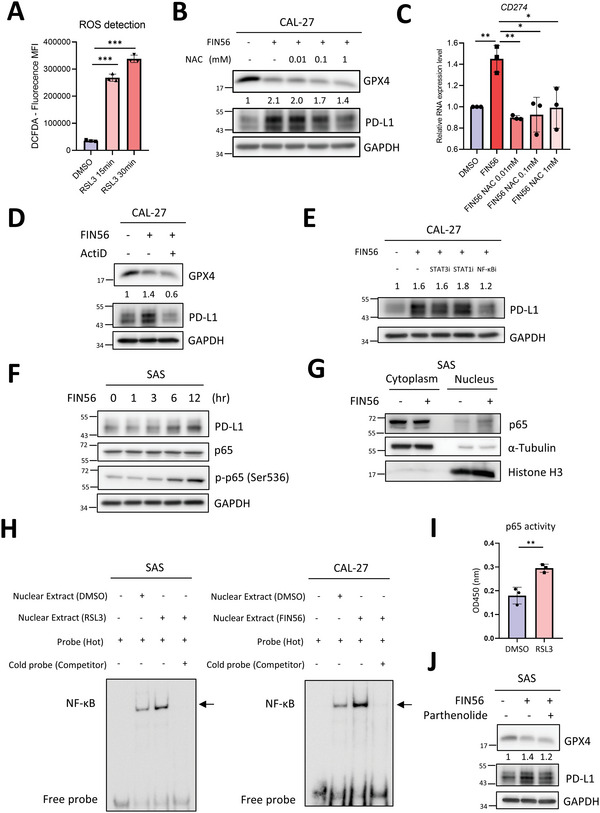
The ferroptosis inducer upregulates PD‐L1 expression in HNSCC through NF‐*κ*B signal pathway. A) Mean fluorescent intensity of DCFDA in CAL‐27 cells treated with RSL3 for 15 min and 30 min. The data is presented in mean ± S.D, *n* = 3. ***p < 0.001 (Student's *t*‐test). B) Western blots to examine GPX4 and PD‐L1 in CAL‐27 cells treated with FIN56 1 µm for 24 h or vehicle control with/without an ROS scavenger *N*‐acetylcysteine (NAC) 0.01, 0.1, or 1 mm for 24 h. GAPDH was a loading control. C) RT‐qPCR to examine the relative mRNA level of *CD274* in CAL‐27 cells treated with FIN56 1 µm for 24 h or a vehicle control with/without NAC 0.01, 0.1, or 1 mm for 24 h. The data is presented in mean ± S.D, *n* = 3. *p < 0.05, **p < 0.01 (Student's *t*‐test). D) Western blots to examine the expression of GPX4 and PD‐L1 in CAL‐27 cells treated with FIN56 1 µm for 24 h or a vehicle control with/without a transcriptional inhibitor actinomycin D (ActiD) 50 nm for 24 h. GAPDH was a loading control. E) Western blots to examine PD‐L1 expression in CAL‐27 cells treated with FIN56 1 µm for 24 h or a vehicle control with/without the indicated inhibitors STAT3i Stattic 1 µm for 24 h, STAT1i Fludabine 1 µm for 24 h, and NF‐*κ*Bi Parthenolide 0.5 µm for 24 h. GAPDH was a loading control. F) Western blots of PD‐L1, total p65, and serine 536 phosphorylated p65 in SAS cells treated with FIN56 1 µm for the indicated durations. GAPDH was a loading control. G) Nucleus‐cytoplasm fractionation and western blots for examining nuclear and cytoplasmic p65. *α*‐tubulin was a control for the cytoplasmic protein and Histone 3 was a control for the nuclear protein. H) Electrophoretic mobility shift assay. Left, nuclear extracts from SAS cells treated with RSL3 1 µm for 24 h or vehicle control. Right, the nuclear extracts of CAL‐27 cells treated with FIN56 1 µm for 24 h or a vehicle control were hybridized with the labeled probe containing an NF‐*κ*B binding element and subject to analysis. The unlabeled probe (cold probe) was used for competition. The shifted bands are indicated. I) The p65 activity assay. Cell extracts from SAS cells treated with RSL3 1 µm for 24 h or a vehicle control were analyzed by NF‐*κ*B  ELISA kit and detected at absorbance 450 nm. The data is presented in mean ± S.D, *n* = 3. **p < 0.01 (Student's *t*‐test). J) Western blots to examine the expression of GPX4 and PD‐L1 in SAS cells treated with FIN56 1 µm for 24 h or a vehicle control with/without cotreatment of an NF‐*κ*B inhibitor Parthenolide 1.5 µm for 24 h. GAPDH was a loading control.

Next, we sought the pathway(s) that mediate ferroptosis‐induced up‐regulation of PD‐L1. The cellular ROS level was significantly increased after RSL3 treatment, as expected (**Figure** **4**A). The ROS scavenger *N*‐acetyl cysteine (NAC) attenuated the RSL3 treatment‐induced protein and mRNA expression of PD‐L1 in CAL‐27 cells (Figure 4B,C). The transcription inhibitor actinomycin D repressed ferroptosis‐induced upregulation of PD‐L1 (Figure 4D), indicating the potential for ferroptosis‐induced expression of PD‐L1 through ROS‐mediated transcriptional activation of *CD274*. Next, we evaluated the possible ferroptotic ROS‐activated pathways in HNSCC cells by co‐treatment with FIN56 and different inhibitors for inflammatory signal pathways, including a STAT3 inhibitor, a STAT1 inhibitor, and an NF‐*κ*B inhibitor. Suppression of NF‐*κ*B but not STAT1 or STAT3 attenuated ferroptosis‐induced PD‐L1 expression (Figure 4E). Analysis of the RNA sequencing data of HSC‐3 cells treated with/without FIN56 showed that NF‐*κ*B signal pathway was one of the major pathways in the analysis of the MSigDB hallmark pathways and the KEGG pathways (Figure S7A,B, Supporting Information). Next, we examined whether ferroptosis activated NF‐*κ*B signaling. Increased phosphorylation and nuclear translocation of p65 was observed in SAS cells under FIN56 treatment (Figure 4F,G). Direct activation of NF‐*κ*B signal by phorbol myristate acetate (PMA) upregulated PD‐L1 in SAS cells (Figure S7C,D, Supporting Information). Activation of NF‐*κ*B by ferroptosis inducers was validated by EMSA (Figure 4H) and ELISA (Figure 4I) in HNSCC cell lines. Inhibition of NF‐*κ*B by parthenolide downregulated FIN56‐induced PD‐L1 expression (Figure 4J). The above data indicate that ferroptosis‐induced ROS activates NF‐*κ*B to up‐regulate PD‐L1 in HNSCC.

Previous studies showed that calcium signal is a character of ferroptosis and a major signal in ROS induction.^[^ ^30^, ^31^ ^]^ We examined whether the calcium signal is also involved in the regulation of ferroptosis‐induced PD‐L1. Treatment of ferroptosis inducers did not induce significant calcium influx or change the pattern of calcium mobilization induced by a calcium ionophore A23187 within 10 min in HNSCC cells (Figure S8A,B, Supporting Information). However, a gradual elevation of cytosolic calcium was detected after hours of FIN56 treatment (Figure S8C, Supporting Information). Nevertheless, FIN56‐induced calcium influx was abrogated in HNSCC cell cultivated with the calcium‐free medium or intracellular calcium chelator BAPTA treatment (Figure S8D,E, Supporting Information). Prolonged treatment with FIN56 (24 h) significantly reduced A23187‐induced calcium mobilization (Figure S8F, Supporting Information). In contrast, prolonged FIN56 incubation did not affect the calcium mobilization induced by an ER calcium ATPase inhibitor thapsigargin (Figure S8G, Supporting Information). Treatment with calcium chelators EGTA‐AM or BAPTA attenuated FIN56‐induced PD‐L1 expression (Figure S8H, Supporting Information), which validates the role of ferroptosis‐induced calcium influx in PD‐L1 upregulation. The above results indicate that extracellular calcium is responsible for ferroptosis‐induced calcium influx, and the contribution of ER‐stored calcium in ferroptosis‐induced calcium influx is yet to be determined.

### 2.5 Ferroptosis Inducers Suppress HNSCC Growth, Modulate Tumor Microenvironments, and Sensitize Murine HNSCC to Anti‐PD‐L1 Antibody Treatment

**Figure 5:**
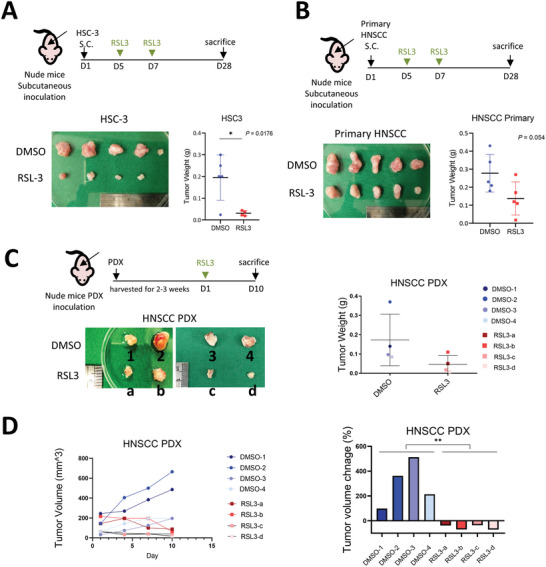
Ferroptosis inducers inhibit the growth of HNSCC tumors. A) Upper, a schema to illustrate the experiment. 1 × 10^6^ of HSC‐3 cells were inoculated into the subcutaneous region of the nude mice. Intratumoral injection of RSL‐3 (100 mg kg^−1^) was administered on the fifth and seventh day. Mice were sacrificed on the 28th day and tumors were harvested. Left lower, the photo of the tumors harvested. Right lower, the tumor weight of the two groups. DMSO group *n* = 5, RSL3 group *n* = 4. The data is presented in mean ± S.D; *p < 0.05 (Student's *t*‐test). B) Upper, a schema to illustrate the experiment. 1 × 10^6^ of the primary HNSCC cells were inoculated into the subcutaneous region of the nude mice. Intratumoral injection of RSL‐3 (100 mg kg^−1^) was given on 5th and 7th day. The mice were sacrificed on the 28th day and tumors were harvested. Left lower, the photo of harvested tumors. Right lower, the tumor weight of the two groups. DMSO group *n* = 5, RSL3 group *n* = 5. The data is presented in mean ± S.D (Student's *t*‐test). C) Upper left, a schema for illustrating the experiment. Patient‐derived xenograft tumors (PDX) were inoculated to the subcutaneous region of the nude mice. Intratumoral injection of RSL‐3 (100 mg kg^−1^) or DMSO control was administered on day1. The mice were sacrificed on the day10 and the tumors were harvested. Lower left, photo of harvested tumors. Right, the tumor weight of the two groups. DMSO group *n* = 4, RSL3 group *n* = 4. The data is presented in mean ± S.D (Student's *t*‐test). D) Left, the tumor volume at day1, day4, day7, and day10. Right, a waterfall plot to show the tumor volume change by quantifying the ratio of tumor volume at the day1 to the day10. DMSO group *n* = 4, RSL3 group *n* = 4. **p < 0.01 (Student's *t*‐test).

We validated the antitumor activity of the ferroptosis inducers in three xenografted HNSCC models. Both the human HNSCC cell line HSC‐3‐formed tumors and primary HNSCC culture‐formed tumors were repressed by RSL3 treatment (**Figure** **5**A,B). RSL3 also inhibited the growth of four patient‐derived HNSCC xenografted tumors (PDX) (Figure 5C,D).

**Figure 6:**
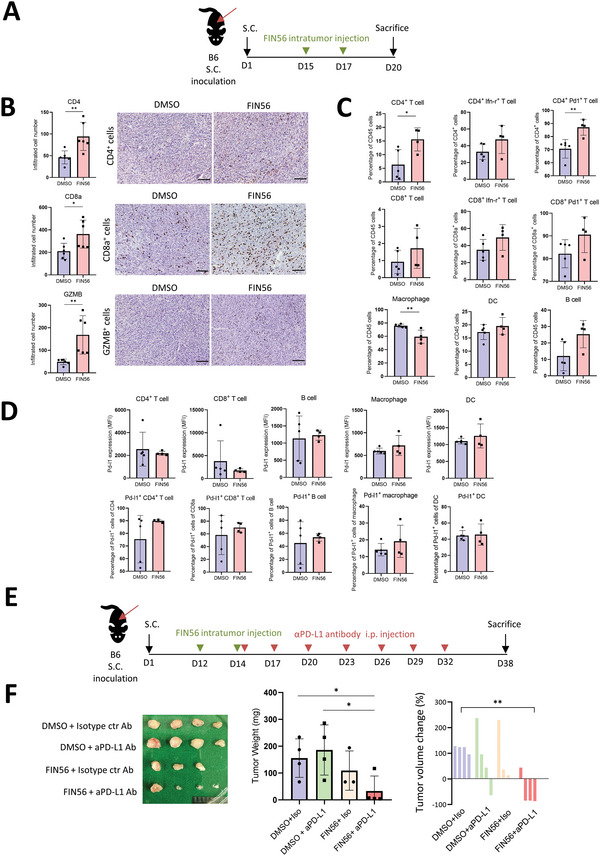
Ferroptosis induced active‐immune TME and sensitizes the tumors to *α*PD‐L1 treatment. A) A schema to illustrate the experiment. 1 × 10^6^ of MTCQ1‐2 cells were inoculated into the subcutaneous region of the B6 mice. Intratumoral injection of FIN56 (100 mg kg^−1^) was administered on the 15th day and 17th day. Mice were sacrificed on the 20th day and tumors were harvested. B) Ferroptotic inducer treated HNSCC tumor tissues were collected for IHC staining. Left, the average infiltrated cells numbers were counted (*n* = 6). The data is presented in mean ± S.D; *p < 0.05, **p < 0.01 (Student's *t*‐test). Right, representative images of the IHC were shown. Scale bar, 100 µm. C) Tumor‐infiltrated CD45^+^ immune cells were assessed by flow cytometry. Population of CD4^+^ T cells, CD8+ T cell, macrophage, DC, and B cell in tumors from the indicated groups. DMSO (*n* = 5), FIN56 (*n* = 4). The data is presented in mean ± S.D: *p < 0.05, **p < 0.01 (Student's *t*‐test). D) Pd‐l1 expression and percentage of pd‐l1^+^ cells of CD4^+^ T cells, CD8^+^ T cell, macrophage, DC, and B cell in tumors from the indicated groups. DMSO (*n* = 5), FIN56 (*n* = 4). The data is presented in mean ± S.D (Student's *t*‐test). E) A schema of syngeneic tumors 1 × 10^7^ MOCL2‐1 cells were subcutaneously injected into the flank of C57BL/6 mice. Tumor‐bearing mice received two intra‐tumor injection of 100 mg kg^−1^ FIN56 on the 12th and 14th day after tumor cell injection. On the 14th day 100 µg anti‐PD‐L1 antibody (Bio X Cell) was administered intraperitoneally to each mouse. Antibodies were administered every 3 days until the 32nd day. The mice were sacrificed on the 38th day post‐tumor cell injection. F) Left, photo of the harvested tumors. Middle, quantification of tumor weight. DMSO with isotype antibody control (*n* = 4), DMSO with anti‐PD‐L1 antibody (*n* = 4), FIN56 with isotype antibody control (*n* = 3), FIN56 with anti‐PD‐L1 antibody (*n* = 4). The data is presented in mean ± S.D; *p < 0.05 (Student's *t*‐test). Right, a waterfall plot to show the tumor volume change by quantifying the ratio of tumor volume at the 38th day to the 12th day. **p < 0.01 (Student's *t*‐test).

We next investigated the influence of the ferroptosis inducer treatment on tumor microenvironments. The murine syngeneic HNSCC model by inoculating the murine HNSCC cell line MTCQ1‐2^[^ ^32^ ^]^ to the subcutaneous region of C57BL/6J mice was applied (**Figure** **6**A). Immunohistochemical staining of the harvested tumors demonstrated the increased infiltration of CD4^+^ cells, CD8^+^ cells, and granzyme B^+^ cells in the FIN56‐treated tumors (Figure 6B). Flow cytometric analysis of the tumor‐infiltrated immune cells revealed an increased CD4^+^ cells, CD4^+^Pd1^+^ cells, a reduced CD11b^+^F4/80^+^ macrophages, and a trend of increased CD8^+^ cells, CD8^+^Ifn*γ* ^+^ cells, CD8^+^Pd1^+^ cells, CD4^+^Ifn*γ* ^+^ cells, and B cells in FIN56‐treated tumors (Figure 6C). For ferroptotic stress upregulates PD‐L1 in HNSCC cells (Figures 3, 4), we examined the influence ferroptotic stress on Pd‐l1 expression of mouse immune cells. The mean fluorescent intensity and the Pd‐l1‐positive subpopulations were not different among the FIN56‐treated and control groups of the syngeneic murine HNSCC tumors (Figure 6D).

Next, we examine the combination of the ferroptosis inducer with an anti‐PD‐L1 antibody (*α*PD‐L1) in a syngeneic murine HNSCC model MOCL2 cell line/C57BL6/J mice.^[^ ^33^ ^]^ Activation of NF‐*κ*B and induction of PD‐L1 expression by sublethal FIN56 was validated in MOCL‐2 cells in vitro (Figure S9A–C, Supporting Information). Upregulated PD‐L1 expression in MOCL2‐formed tumors under FIN56 treatment was observed (Figure S9D, Supporting Information). We further treated tumor‐bearing mice with *α*PD‐L1, FIN56, a combination of *α*PD‐L1 and FIN56, or an IgG isotype control. The *α*PD‐L1 treatment did not show a significant suppressive effect on syngeneic MOCL2 tumors, and modest tumor suppression was observed in the FIN56 group. The combination of *α*PD‐L1 and FIN56 revealed the highest tumor suppression among the four groups (Figure 6E,F). In summary, the results indicate that ferroptosis inducers inhibit HNSCC growth, influence the infiltrated immune cells and demonstrate a combinatory effect with the *α*PD‐L1 treatment.

### 2.6 The Ferroptosis Signature Correlates with an Immune‐Active State of HNSCC

We investigated the relevance of the ferroptosis signature to the immune environment of HNSCC. We first applied the TCGA‐HNSC data set to investigate the correlation between ferroptosis signature/*GPX4* and T cell infiltration/activation. A higher expression of *CD4*, *CD8A*, *CD3D*, *CD28*, *GZMB*, *IFNG*, *PRF1*, and *IL2* was observed in the ferroptosis_high patient group (Figure S10A, Supporting Information). A positive correlation was shown between the ferroptosis signature and the CD8^+^ cytotoxic T cell signature (Figure S10B, Supporting Information). Further analysis by TIMER 2.0 demonstrated that *GPX4* expression was negatively correlated with the level of CD8 T cell infiltration in most analyzes for HPV‐negative patients, while a positive association between *GPX4* and T cell infiltration was noted in most analyses for HPV‐positive patients (Figure S10C, Supporting Information). The correlation between individual genes showed a distinct correlation pattern of correlation in the HPV negative and positive subgroups. The T‐cell infiltration score was significantly higher in the HPV positive group compared to the HPV‐negative patients (Figure S10D, Supporting Information). In HPV‐negative HNSCC, there was a trend that *GPX4* negatively correlated with *CD8A* expression and significantly correlated with the T cell activation marker *IFNG* or *GZMB* negatively. On the contrary, the HPV positive patient group exhibited a trend of positive correlation between *GPX* and T cell activation markers, and *GZMB* reached statistical significance (Figure S10E, Supporting Information).

**Figure 7:**
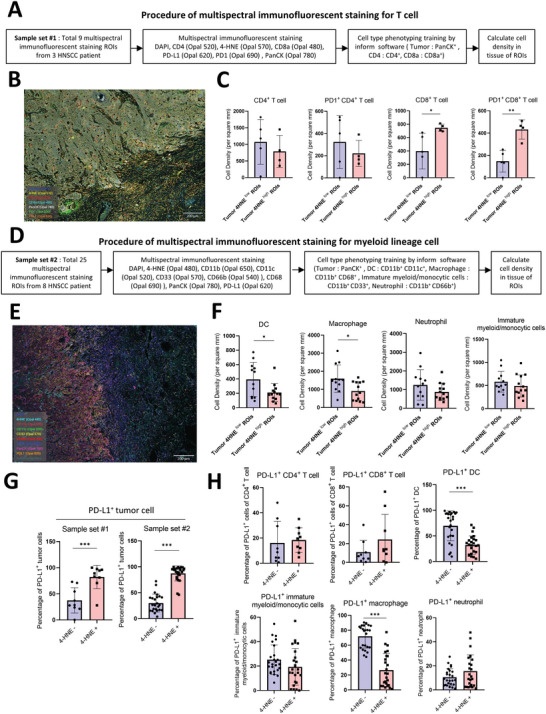
The ferroptosis signature correlates with an active immune environment in HNSCC patient samples. A) Flowchart of the multispectral immunofluorescent imaging of sample set #1 for T cell. B) Representative multispectral immunofluorescent images of sample set #1 for T cell. Scale bar = 200 µm. C) Cell density of CD4^+^ T cell, CD4^+^PD1^+^ T cell, CD8^+^ T cell, CD8^+^PD1^+^ T cell in tumor 4‐HNE^high^ (*n* = 4) or 4‐HNE^low^ (*n* = 5) ROIs. The data is presented in mean ± S.D. *p < 0.05, **p < 0.01 (Student's *t*‐test). D) Flowchart of the multispectral immunofluorescent imaging of sample set #2 for myeloid lineage cell. E) Representative multispectral immunofluorescent images of sample set #2 for myeloid lineage cell. Scale bar = 200 µm. F) Cell density of DC, macrophage, neutrophil, and immature myeloid/monocytic cells in tumor 4‐HNE^high^ (*n* = 13) or 4‐HNE^low^ (*n* = 12) ROIs. The data is presented in mean ± S.D. *p < 0.05 (Student's *t*‐test). G) Percentage of PD‐L1^+^ cells of 4‐HNE^−^ tumor cells or 4‐HNE^+^ tumor cells in sample set#1 (*n* = 9) and sample set#2 (*n* = 25) ROIs. The data is presented in mean ± S.D. ***p < 0.001 (Student's *t*‐test). H) Percentage of PD‐L1^+^ cells of 4‐HNE^−^ or 4‐HNE^+^ CD4^+^ T cell, CD8^+^ T cell in sample set#1 (*n* = 9) ROIs. Percentage of PD‐L1^+^ cells of 4‐HNE^−^ or 4‐HNE^+^ DC, macrophage, neutrophil, and immature myeloid/monocytic cells in sample set#2 (*n* = 25) ROIs. The data is presented in mean ± S.D. ***p < 0.001 (Student's *t*‐test).

Next, we applied multispectral immunofluorescent imaging to investigate the correlation between ferroptotic stress and immune microenvironment in the HNSCC sample. The lipid peroxidation marker 4‐hydroxynonenal (4‐HNE) was applied to indicate the ferroptotic stress in HNSCC samples. We conducted two sets of samples for investigation of T cells (set#1) (**Figure** **7**A–C) and myeloid linage cells (set#2) (Figure 7D–F). The workflow is illustrated in Figure 7A,D, and the representative multispectral immunofluorescent images are shown in Figure 7B,E. A significant higher density of CD8^+^ cell and CD8^+^PD1^+^ cells was demonstrated in ROIs with higher 4‐HNE in tumor cells (Figure 7C). A lower density of DC and macrophages was shown in ROIs with higher 4‐HNE in tumor cells (Figure 7F). Among the myeloid immune cell types we screened, macrophages were the dominant PD‐L1 expressing cell (Figure S11, Supporting Information). We also examined the PD‐L1 expression in tumor and immune cells of the ROIs with high versus low 4‐HNE signals. An increased PD‐L1 expression was noted in tumor cells of ROIs with high 4‐HNE (Figure 7G). In contrast, a reduced percentage of PD‐L1^+^ DC and macrophages was shown in ROIs with high 4‐HNE (Figure 7H).

**Figure 8:**
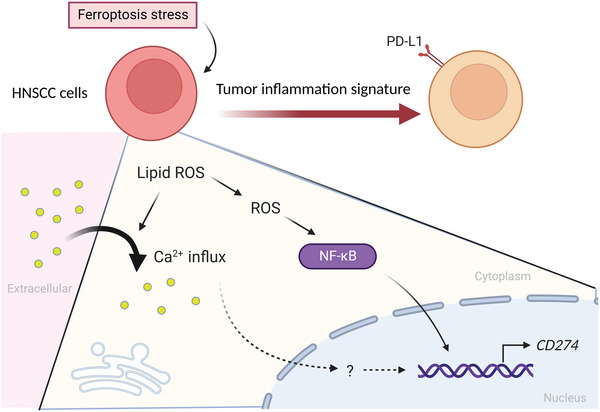
The model of ferroptosis activated signal and signature of HNSCC. A schema to summarize the finding in this study. When HNSCC cells are under ferroptosis stress, lipid ROS‐activated NF‐*κ*B and increased intracellular calcium signal contribute to up‐regulation of *CD274* expression. The ferroptosis signature correlates with an active immune environment of HNSCC. Summary picture was created with Biorender.

We summarize the main findings of this study in **Figure** **8**. In HNSCC, ferroptotic stress generates lipid ROS to activates NF‐*κ*B, which subsequently activates *CD274* transcription. Furthermore, ferroptosis‐induced calcium signaling also contributes to the up‐regulation of PD‐L1. HNSCC cells with ferroptosis signature reveal an inflamed phenotype together with PD‐L1 expression, indicating the potential of priming HNSCC with ferroptosis inducers to improve the efficacy of *α*PD‐L1.

## 3 Discussion

The antitumor effect of ferroptosis has been extensively investigated, especially in drug‐resistant mesenchymal‐like cancer stem cells.^[^ ^4^, ^5^, ^6^, ^7^ ^]^ Here, we validated the antitumor activities of ferroptosis inducers in vivo. In addition to the cytotoxic activities of ferroptosis inducers, ferroptosis has been found to induce immunogenic cell death with the release of immunoregulating molecules such as HMGB1^[^ ^34^ ^]^ and ATP.^[^ ^35^ ^]^ Ferroptotic cancer cells modulate tumor microenvironments to an immunoactive state.^[^ ^36^ ^]^ In this study, we observed that ferroptotic stress modulates tumor‐infiltrated immunocytes in murine model and HNSCC samples. On the basis of the evidence presented above, we suggest that ferroptosis inducers suppress tumor growth through a direct tumor suppressing effect and microenvironmental modulation.

Several strategies have been reported to prime the tumor to facilitate the effectiveness of ICIs, including chemotherapy,^[^ ^37^, ^38^ ^]^ toll‐like receptor agonist,^[^ ^39^ ^]^ STING1 agonist,^[^ ^40^ ^]^ and radiotherapy.^[^ ^41^ ^]^ The main finding of our study is that ferroptotic stress in HNSCC cells upregulates PD‐L1, which provides the potential to prime HNSCC to potentiate the efficacy of ICIs. The combinatory effect of ferroptosis inducers and anti‐PD‐L1 antibody was validated in the syngeneic murine model of this study. Intriguingly, a recent study demonstrated that immunotherapy‐activated CD8^+^ T cells enhance ferroptosis in tumor cells, which contributes to the anti‐tumor efficacy of immunotherapy.^[^ ^42^ ^]^ This finding together with our result strengthen the potential synergy of ferroptosis inducer and immunotherapy. Therefore, the development of optimal‐dosed ferroptosis inducer as a priming strategy for augmenting antitumor immune response is a promising strategy for cancer immunotherapy. An interesting question is whether ferroptotic stress in tumor cells affects PD‐L1 expression in immunocytes. Recent studies have shown that ferroptosis affects macrophage infiltration and polarization in the murine liver cancer model.^[^ ^43^ ^]^ However, we did not observe that ferroptotic stress in HNSCC cells influences PD‐L1 expression in immunocytes in our study. The possible explanation is that our study observed ferroptotic stress in tumor cells rather than directly in immune cells, and the distinct signaling pathways between tumor cells and immune cells should also be considered. Further investigation of ferroptotic stress in infiltrated immune cells of different tumors is mandatory.

In this study, we found that both ROS‐driven NF‐*κ*B activation and calcium influx contribute to ferroptosis‐induced PD‐L1 expression. ROS has been well known to activate NF‐*κ*B signaling,^[^ ^44^ ^]^ and PD‐L1 is one of the major immune‐related molecules upregulated by NF‐*κ*B pathway.^[^ ^45^ ^]^ Besides, the increased intracellular calcium signal is a common response to stress.^[^ ^46^ ^]^ Here we showed that prolonged ferroptotic stress induced calcium influx after hours, which is consistent with the previous finding that ferroptosis caused a sustained increase in cytosolic calcium that is related to plasma membrane damage rather than ion channel dependent.^[^ ^30^ ^]^ There are other sources of calcium ion storage in cells such as the ER^[^ ^47^ ^]^ and mitochondria^[^ ^48^ ^]^ which may contribute to the increase in cytosolic calcium. Our result indicates that extracellular calcium is the main source of ferroptosis‐induced calcium influx, and the role of intracellular calcium storage organelles in the ferroptosis‐induced calcium signal appears to be minor and remains not yet fully determined.

Another interesting finding of the study is that the ferroptosis‐induced inflammation signature or PD‐L1 expression is more prominent in the HPV‐negative HNSCC group than in the HPV‐positive group. HPV‐positive HNSCC is generally considered an inflamed tumor with an immune‐activated microenvironment compared to HPV‐negative patients.^[^ ^49^ ^]^ Our study implies that in patients with HPV negative HNSCC, analysis of the ferroptosis signature may be helpful to categorize patients as immune active and immune inactive, which will be informative in selecting the appropriate cases for ICI therapy. It may also indicate the potential of the application of ferroptosis‐inducing agents in HPV‐negative HNSCC to create an immune‐active environment. A larger cohort with additional modalities is mandatory to validate the relationship between the ferroptosis signature and HPV‐negative HNSCC.

In summary, our study demonstrates spatial correlation and signal linkage between ferroptosis and PD‐L1 in HNSCC. We also show that ferroptosis induces the expression of tumoral PD‐L1 through the membrane damage‐independent (NF‐*κ*B pathway) and dependent (calcium influx) mechanisms. Furthermore, this study also highlights the potential to prime advanced HNSCC with ferroptosis inducers prior to the treatment of ICIs. The optimization of the formulation and delivery routes of ferroptosis inducers will be crucial for the future application of ferroptosis inducers in clinical cancer treatment.

## 4 Experimental Section

### HNSCC Samples

The study was approved by the Institutional Review Board of Taipei Veterans General Hospital (IRB certificate No. 2017‐05‐013AC) and Taichung Veterans General Hospital (IRB certificate No. CE22076A).The experiments were carried out with the full, informed consent of the subjects. Six independent sets of samples were used for the experiments. The characteristics are presented in Table S1, Supporting Information. The first group comprised 65 dissected tumor samples from 21 patients with HNSCC and contralateral normal oral epithelium. Bulk RNA sequencing was performed for tumors and adjacent normal tissues. The second group comprised 3 primary HNSCC tumors from different patients for scRNA‐seq. The third group consisted of 2 HNSCC patient samples for Visium spatial transcriptomic analysis (eight slides). The fourth group comprised samples from 91 patients with HNSCC for tissue array IHC staining. The fifth group consisted of samples from 2 patients with HNSCC for PDX. The sixth group consisted of samples from 11 patients with HNSCC with a total of 34 region of interests (ROIs) for multispectral immunofluorescent staining.

### Bulk RNA Sequencing

The matched pairs of primary HNSCC tumors (including invasive front and intratumor core regions), metastatic tumors, and adjacent normal tissues from patients with HNSCC at Taipei Veterans General Hospital (TVGH) were subjected to RNA sequencing analysis (Figure S1A, Supporting Information). The samples were frozen in RNA Later Tissue Storage Reagent (AM7021, Invitrogen) and stored at −80 °C. RNA was harvested using TRIzol reagent (15596026, Invitrogen). RNA quality check was measured by bioanalyzer (RIN > 1.5). The RNA QC library information is listed in Table S9, Supporting Information. An Illumina TruSeq RNA Sample Prep Kit was used with 1 µg of total RNA for the construction. After reverse transcription, cDNA sequencing was performed using the Illumina NextSeq500 and Illumina HiSeq2500 (Illumina, Inc.). Reads were trimmed and clipped for quality control in AfterQC v0.9.7. and read quality was checked for each sample using FastQC v0.11.7. The high‐quality reads were then imported into STAR v2.6.1a for alignment in BAM files. BAM files were imported into RSEM v1.3.1 for expected counts, FPKM, and TPM estimation. The expected count table was extracted from each RSEM output gene result file and also imported into DESeq2 v1.26.0 for the rlog transformation. The GRCh38.94 reference genome was used for gene alignment.

### Single‐Cell RNA Sequencing

To obtain cells for scRNA‐seq analysis, the HNSCC tumors were dissociated using a tumor dissociation kit (Miltenyi Biotec), and dead cells were removed using a dead cell removal kit (MACS). Single cell suspension from tumors with viability greater than 80% was loaded onto the 10x Genomics Next GEM Chip G for the target capture of ≈8000 cells/chip. For library construction and sequencing, droplet‐based scRNA‐seq (10x Genomics Chromium Single Cell 3’ Reagent Kit v3.1 no. 1000121) was used for single‐cell library preparation. After reverse transcription, cDNA sequencing was performed using the Illumina Novaseq 6000 (Illumina, Inc.). scRNA‐seq reads were processed using a 10x Genomics Cell Ranger pipeline (version 6.1.1, 10x Genomics). Chemistry Batch Correction algorithm (Cell Ranger v3) was used to correct batch effects between samples. The algorithm is based on mutual nearest neighbors (MNN) to identify similar cell subpopulation between batches. Then data were analyzed by 10× Loupe Browser (version 6.2, 10x Genomics). The clustering of cells in the dataset was performed using a Uniform Manifold Approximation and Projection (UMAP) algorithm in Loupe Browser. GO analysis of differentially expressed genes between clusters was performed using the online Enricher website (https://maayanlab.cloud/Enrichr/).

### Tissue Processing and Data Generation for Visium Spatial Gene Expression

A total of eight samples obtained from primary tumors of two HNSCC patients were subjected to Visium spatial transcriptomics analysis. The patient characteristics are illustrated in Table S1, Supporting Information. The preparation of the tissue section followed the Visium Spatial Tissue Optimization User Guide Rev D (10x Genomics, CG000239). In brief, frozen samples were cryosectioned on Visium Tissue Optimization Slides. Histology images were taken with an Olympus IX83 (10× PH Objective). The library construction was performed according to the Visium Spatial Gene Expression User Guide. Libraries were loaded at 250 pM and sequenced on a NovaSeq 6000 System (Illumina) at a sequencing depth of 0.05 M reads/spot following the formula provided by the manufacturer (10x Genomics). Data processing of Visium data, raw FASTQ files and images was performed with Space Ranger software (Version 1.2.1, 10x Genomics) and the GRCh38 v98 reference genome was used for gene alignment. Analysis ferroptosis‐related genes and the visualization of the images were performed using Loupe Browser (version 6.2, 10x Genomics).

### Weighted Correlation Network Analysis

To explore the biological characteristics of the heterogeneity of the inner tumor core, invasive fronts, and metastatic tumors of the HNSCC transcriptome, weighted correlation network analysis (WGCNA) was implemented with the R WGCNA package (v. 1.70‐3 in R 4.0.3)^[^ ^50^ ^]^ using log2 (TPM + 1) normalized RNA‐seq values for 18 428 protein‐coding genes in 65 samples from HNSCC patients (Figure S1B, Supporting Information). Protein‐coding genes with 90% of zero values among 65 samples were excluded from the analysis. A soft thresholding power of 17 was selected to maximize the scale‐free topology model fit as it plateaued above 0.7. For module identification, the “cutreeDynamic” function was performed based on the “tree‐cutting” algorithm with deepSplit = 2 and minModulesize = 5. The modules were characterized by Gene Ontology (GO) biological process (BP), GO cellular component (CC), and KEGG pathway terms using the “enrichGO” and “enrichKEGG” functions in the R ClusterProfiler package (v. 3.18.1 in R 4.0.3).^[^ ^23^ ^]^ To provide a concise representation, the network was trimmed by preserving only the genes that belonged to representatively enriched terms (one of the top 20; FDR‐*q* value ≤ 0.05) from each annotation category, edges with correlation weight greater than 0.01 (positive associations only) and keeping at most the top 50 edges for each source and target node. To quantify differential patterns of a specific signature‐related subnetwork among three parts of the tumors, a further functional annotation of the modules was performed with a hypergeometric test using ferroptosis, inflammation, tumor inflammation, EMT, and interferon‐stimulated gene (ISG) signature sets (Figure 1A; Figure S1B, Supporting Information).^[^ ^24^, ^25^, ^26^, ^27^ ^]^ The modules were then integrated, which had a specific enriched signature (FDR‐*q* value ≤ 0.05) and at least one of the signature genes, with all their neighboring genes in the network to determine the signature‐related subnetwork (Figure 1A–D; Figure S1C,D, Supporting Information). For example, there were 42, 63, 132, 116, and 66 genes in the ferroptosis, inflammation, tumor inflammation, EMT, and ISG signature‐related subnetworks, respectively. For each signature‐related subnetwork, the log_2_ T/N values (i.e., fold changes) of all the genes between tumors (T) from IC, IF, or M sites and corresponding normal tissues (N) were evaluated. Finally, a two‐sided Wilcoxon signed‐rank test was used to statistically quantify the differential patterns of subnetworks among three parts of the tumor (Figure S1E, Supporting Information). Here, the differential patterns of each subnetwork that satisfied the following criteria were defined: i) the median of log_2_ T/N values among all genes from IC to IF to M gradually increase; ii) *p*‐value ≤ 0.05 between IC and IF; and iii) *p*‐value ≤ 0.05 between IC and M. The correlation networks/subnetworks with modules were visualized in an open‐source software platform, Cytoscape (version 3.8.2).^[^ ^51^ ^]^

### Animal Experiments

The animal experiment was approved by the Institutional Animal Care and Utilization Committee of Taipei Veterans General Hospital (IACUC certificate No. 2019–040). To evaluate the antitumor effect of the ferroptosis inducer, 1 × 10^6^ HSC3 cells or primary HNSCC cells were subcutaneously injected into the flanks of nude mice. Tumor‐bearing mice received two intratumor injections of 100 mg kg^−1^ RSL3 on the 5th and 7th days after tumor cells. The mice were sacrificed on the 28th day post‐tumor‐cell injection. For patient‐derived xenografts (PDX), the HNSCC specimens were first rinsed twice and immersed in Matrigel (Becton‐Dickinson) at 37 °C. The tumors were cut into 1 mm^3^ pieces and subcutaneously implanted in 4 weeks old female nude mice to establish PDX. For investigation of the ferroptotic drug‐induced immune cell infiltration in the syngeneic HNSCC mouse model, 1 × 10^6^ MTCQ1‐2 cells were subcutaneously injected into the flanks of C57BL/6 mice. The tumor‐bearing mice received two intratumor injections of 100 mg kg^−1^ FIN56 on the 15th and 17th days after inoculation of tumor cells. The mice were sacrificed on the 20th day post tumor cell injection and tumor sample were collected for IHC and immunophenotyping of infiltrated immune cells. For investigation of the anti‐tumor effect of the ferroptotic inducer combined with an anti‐PD‐L1 antibody in the syngeneic HNSCC mouse model, 1 × 10^7^ MOCL2‐1 cells were subcutaneously injected into the flanks of C57BL/6 mice. The tumor‐bearing mice received two intratumor injections of 100 mg kg^−1^ FIN56 on the 12th and 14th days after tumor cell injection. On the 14th day, 100 µg anti‐PD‐L1 antibody (Bio X Cell) was administered intraperitoneally to each mouse. Antibodies were administered every 3 days until the 32nd day. The mice were sacrificed on the 38th day post‐tumor‐cell injection. The volume of the tumors was measured regularly and the weight of the tumors was measured after they were harvested.

### Statistical Analysis

Statistical analyzes were performed using GraphPad Prism 8 (GraphPad Software). The two‐sided independent Student's *t‐*test was used to compare continuous variables between two groups. The Pearson correlation test was used to analyze the correlation between two continuous factors. All statistical data were derived from at least three independent biological replicates, and each experiment contained at least two technical replicates. *p* ≤ 0.05 was considered statistically significant (*: ≤0.05, **: ≤0.01, ***: ≤0.001).

### Data and Code Availability

The accession numbers for the data reported in this paper are GEO GSE178537 (review token: mnytwiwedxkfvsf), GSE181300 (review token: wjuhsswmvnylxub), GSE185965 (review token: krytcgkgflifjyt), and GSE196947 (review token: wxipsesoftkdhoz).

## Conflict of Interest

The authors declare no conflict of interest.

## Author Contributions

M.‐H.Y. supervised the entire study. M.‐H.Y. and C.‐H.C. conceived and designed the experiments. C.‐H.C. performed most of the experiments with the help of C.‐Y.C. and Y.‐W.C. C.‐Y.L. analyzed the bulk RNA‐seq data, and C.‐W.H. contributed to the analysis under the supervision of C.‐Y.L. M.‐H.Y., C.‐C.W., S.‐K.T., and P.‐Y.C. provided clinical samples, patient care, and collected demographic data. C.‐H.C. and M.‐H.Y. wrote the paper with the help of C.‐Y.L. for the bioinformatics part.

## Supporting information

Supporting Information

Supporting Information

Supporting Information

Supporting Information

Supporting Information

Supporting Information

Supporting Information

Supporting Information

Supporting Information

Supporting Information

## Data Availability

The data that support the findings of this study are available from the corresponding author upon reasonable request.
