# Supplementary material for: Ferroptosis Signature Shapes the Immune Profiles to Enhance the Response to Immune Checkpoint Inhibitors in Head and Neck Cancer
Source: Adv Sci (Weinh). 2023 Apr 7;10(15):2204514. doi: 10.1002/advs.202204514 (PMC10214241; doi:10.1002/advs.202204514)
Supplement: Supplementary file 1 — Supporting Information [file ADVS-10-2204514-s007.pdf]

ADVANCED SCIENCE
Open Access

Supporting Information

for *Adv. Sci.*, DOI 10.1002/advs.202204514

Ferroptosis Signature Shapes the Immune Profiles to Enhance the Response to Immune Checkpoint Inhibitors in Head and Neck Cancer

*Chih-Hung Chung, Chun-Yu Lin, Chih-Yu Chen, Chun-Wei Hsueh, Yao-Wen Chang, Chen-Chi Wang, Pen-Yuan Chu, Shyh-Kuan Tai and Muh-Hwa Yang**

Supporting Information

Supplementary Materials and Methods

Ferroptosis core enrichment gene analysis. To explore the core signature of ferroptosis, we analyzed the top 1500 genes expressed in the high ferroptosis signature of the Visium analysis (Table S6A, Supporting Information) and 539 upregulated genes (FPKM fold change ≥1.5) in FIN56-treated HNSCC HSC-3 cells compared to the control (Table S6B, Supporting Information). The 42 genes upregulated in both datasets (Table S6C, Supporting Information) were subjected to Gene Ontology (GO) analysis via the online website GOnet (https://tools.dice-database.org/GOnet/) (Table S6C and Table S7, Supporting Information).

Cell lines, plasmids, and reagents. Six human HNSCC cell lines (FaDu, SAS, HSC-3, TE1, CAL-27, OECM-1) and the murine oral squamous cell carcinoma cell line MTCQ1 and MOCL2 were used in this study. MTCQ1 and MOCL2 cell lines were provided by Dr. Kuo-Wei Chang (National Yang Ming Chiao Tung University of Taiwan). [32,33] The MTCQ1-2 and MOCL2-1 subline were generated by inoculating the parental cells into the subcutaneous region of C57BL/6J mice to form tumors and cultivating the tumor cells to enhance the in vivo tumorigenicity. RSL3 (#19288), FIN56 (#25180), Ferrostatin-1 (#17729), BODIPY™ 581/591 C11 lipid peroxidation sensor (#27086), BAPTA-AM (#15551), EGTA-AM (#20401), Actinomycin D

(#11421), STAT3 inhibitor Sttattic (#14590), STAT1 inhibitor Fludarabine (#14128), Phorbol 12-myristate 13-acetate (PMA) (#10008014) and NF-κB inhibitor Parthenolide (#70080) were purchased from Cayman Chemical. TransAM® NF-κB activation assays kit (#43296) was purchased from Activemotif. NFκB EMSA Kit (#GS-0030) was purchased from Signosis N-Acetyl-L-cysteine (NAC) (#A7250), WST-1 reagent and Cisplatin were purchased from Sigma-Aldrich. The Fluo-4 AM calcium indicator (HY-101896) was purchased from MCE. The DCFDA cellular ROS detection assay kit (#ab113851) was purchased from Abcam. The 7-AAD staining solution (#ab228563) was purchased from Abcam.

WST-1 cell viability assay. To estimate cell viability, 1×10^4 cells per well were seeded in a 96-well plate, incubated overnight and then treated with various concentrations of reagents. After 24 h, 10 μL/well Cell Proliferation Reagent WST-1 was added and incubated for 1 h at 37 °C and 5% CO_2. The absorbance of the samples against a blank background control was measured using a microplate ELISA reader. The wavelength for measuring the absorbance of the formazan product was 460 nm. The reference wavelength was 600 nm.

Flow cytometry analysis. Cells were harvested and washed twice with PBS. The cells

were then incubated with primary antibodies (listed in Table 8, Supporting Information.) for 1 h at 4 ° C and then with secondary antibodies for 30 min at 4 °C. Cells were washed and resuspended in 200 µl fresh PBS and then immediately analyzed with a flow cytometer (CytoFLEX, Beckman). Gating strategies of the experiments were showed in Figure S12, Supporting Information.

Lipid peroxidation assessment by C11-BODIPY581/591 staining. The HNSCC cell lines FaDu, CAL-27, SAS, OECM-1, HSC-3, and TE-1 and primary HNSCC cells were treated with the GPX4 inhibitor RSL3 at 1 µM or DMSO for 4 h and stained with C11-BODIPY581/591. The fluorescence shifts represent the intensity of the lipid-ROS level. Cells were then collected by trypsinization for staining. For C11-BODIPY581/591 staining, cells were resuspended in PBS containing 5 µM C11-BODIPY581/591 and incubated for 15 min at 37 °C. Cells were washed and resuspended in 200 µl fresh PBS and then analyzed immediately with a flow cytometer (CytoFLEX, Beckman).

Intracellular calcium detection. Intracellular calcium detection was performed using Fluo-4 AM (HY-101896, MedChemEx, Monmouth Junction, NJ, USA). Cells were harvested and washed with PBS. The cells were then incubated with Fluo-4 AM 1µM in PBS for 60 min at 37 ° C. Cells were washed twice with 500 µl PBS and immediately

analyzed with a flow cytometer (CytoFLEX, Beckman).

Calcium mobilization and measurement. Prepared tumor cells ($2x10^6$/ml) were loaded with fluorescent Ca2+ indicator (Fluo-4/AM, 1μM) at 37 ° C for one hour incubation, followed by washing cells with PBS and resuspended in calcium-containing HBSS. For calcium mobilization, Fluo-4-labeled cancer cells ($5x10^6$ /ml) were treated with indicated concentrations of FIN56 (0, 1, 2, 5, 10 μM) for 3 min (the short-time effect) before being challenged with calcium ionophore (A23187, 5μM) or thapsigargin (selective inhibitor of the sarco-endoplasmic reticulum calcium ATPase, 4μM). Cytosolic calcium concentration was measured using a Hitachi F7000 fluorescence spectrophotometer at 37 ° C (excitation 494 nm; emission 516 nm). At the end of the experiment, cells were lysed with Triton X-100 (0.1%) and then addition of EGTA (10 mM) to obtain maximal and minimal fluorescence, respectively. Intracellular calcium concentration was calculated using the dissociation constant (Kd) of Fluo-4/AM (355 nM). The area under the time concentration curve (AUC) was processed by Graphpad software for calcium mobilization. For the long-term effect of FIN56 treatment, cells were cultured and treatments of the FIN56 or DMSO control treatments were administered with the indicated time, followed by the collection of cells as described above for the calcium assay.

Mouse tumor immunophenotyping. Mouse tumor tissues were isolated by mouse tumor dissociation kit (Miltenyi, # 130-096-730) and gentleMACS dissociator. All above reagents instruments were obtained from Miltenyi Biotech and followed the manufacturer's protocols. Tumors tissues were minced into small pieces and incubated in RPMI medium containing digestion enzyme mixture. Next, tissue dissociation by gentleMACS machine (program m_TDK_1) for 45 min at 37 °C. Then cells were filtered with a 70-μm cell strainer and remove RBC by RBC Lysing Buffer (Biolegend) and washed with FACS buffer (phosphate-buffered saline with heat-inactivated 2% FBS and 0.1% sodium azide). Cells were processed for surface marker staining and then intracellular molecule staining. Samples were analyzed with a flow cytometer (CytoFLEX, Beckman). Antibodies used in the experiments are detailed in Table 8, Supporting Information.

Immunohistochemistry (IHC). The IHC experiments were performed using a NovolinkTM polymer detection system kit (# RE7150-K, Lecia Biosystem) according to the manufacturer's protocol. Briefly, formalin-fixed, paraffin-embedded (FFPE) tumor sections (5 μm thick) were deparaffinized and rehydrated, followed by antigen retrieval with citrate sodium buffer (pH=6.0). After washing with TBS-T (0.05%

Tween 20) three times, sample slides were treated with Peroxidase Block and Protein Blocking reagents (Lecia Biosystem) before overnight primary antibody incubation (1:400 for CD8a CST#98941; 1:200 for CD4 CST #25229; 1:200 for GZMB CST #46890; 1:100 for PD-L1 CST #13684; 1:100 for GPX4 ab125066). Next, the slides were incubated with horseradish peroxidase (HRP)-conjugated polymer (30 min, RT), followed by diaminobenzidine (DAB) development and counterstaining with Mayer hematoxylin. Images were captured at 20X magnification using an Olympus microscope system (Olympus BX51; Olympus Corp., Japan).

HNSCC patient-derived xenografts (PDXs). The surgically resected HNSCC patient tumor is cut into 5-10 pieces (2 mm x 2 mm). The number of pieces implanted depends on size of the patient tumor. One tumor piece is implanted per mouse subcutaneously into the left flank of nude mice. Tumor were harvested for 2-3 weeks and performed drug test by intra-tumor injection. Patient PDX case #1 for DMSO-1, DMSO-2, DMSO-3, RSL3-a, RSL3-b. Patient PDX case #2 for DMSO-4, RSL3-c, RSL3-d. The patient information of individual PDX tumor were describe in Table 1, Supporting Information.

Human PBMC isolation. Whole blood was taken from healthy adult volunteer and collected in K2E(EDTA) vacutainer tubes. Peripheral blood mononuclear cells (PBMC)

were isolated from the buffy coat by density gradient centrifugation with Ficoll-Paque PLUS (density 1.077 g/mL), followed by PBS washes until the supernatant was clear. PBMC were suspended in complete media (RPMI supplemented with 10% heat-inactivated FBS, 1% L-glutamine, 1% penicillin-streptomycin). After PBMC treated with feroptotic drugs *in vitro*. PBMC were washed by PBS and treated with Fc blocker and resuspended in FACS buffer for surface marker staining, then followed by flow cytometer analysis. Antibodies used in the experiments are detailed in Table S8, Supporting Information.

Multiplex immunofluorescence staining of HNSCC samples. HNSCC samples were stained by the Opal IHC Kit (Akoya Biosciences) with a multi-color opal panel. Two multi-color opal panel sets were described below: opal panel set#1 for tumor and lymphoid cell were identified by DAPI, CD4 (Thermo, 1:20, Opal 520), 4-HNE (ab46545, 1:250, Opal 570), CD8a (Thermo, 1:50, Opal 480), PD-L1 (CST#13684, 1:100, Opal 620), PD1 (ab137132, 1:250, Opal 690), and PanCK (ab27988, 1:500, Opal 780). Opal panel set#2 for tumor and myeloid cell were identified by DAPI, CD11c (Abcam, 1:500, Opal 520), CD33 (Ab269456, 1:200, Opal 570), 4-HNE (ab46545, 1:250, Opal 480), CD66b (BD555723, 1:500, Opal 540), PD-L1 (CST#13684, 1:100, Opal 620), CD11b (ab52478, 1:1000, Opal 650), CD68 (ab955, 1:100, Opal 690) and

PanCK (ab27988, 1:500, Opal 780). After completion of staining, the Vectra Polaris Automated Quantitative Pathology Imaging System (Akoya Biosciences) was used to scan multispectral data using Phenochart (1.0.12) software (Akoya Biosciences). The inForm software (Ver. 2.6) was applied to further image cell density analysis. Total 34 multispectral images from 12 HNSCC patients were analyzed. Information on the antibodies used in the experiments is listed in Table S8, Supporting Information.

Electrophoretic mobility shift assay. The p65 electrophoretic mobility shift assay (EMSA) was conducted using an NF-κB EMSA Kit (GS-0030, Signosis, Santa Clara, CA, USA). Briefly, the nuclear extract from SAS cells treated with 1μM RSL3 for 24 h was incubated with a p65 binding probe labeled with biotin, and then protein/DNA complexes were separated on a nondenaturing polyacrylamide gel. The gel was transferred to a nylon membrane and detected using a streptavidin-HRP conjugate and a chemiluminescent substrate. The shifted bands corresponding to the protein/DNA complexes were visualized in comparison to the unbound dsDNA. The bands were visualized after exposure to film or a chemiluminescent imaging system.

ELISA for NF-κB activation. NF-κB activation in cells was analyzed by using TransAM® NF-κB/p65 ELISA kits (Active motif, Carlsbad, CA, USA) according to

the manufacturer's instructions. The cell extract from SAS cells was treated with 1 μM RSL3 or DMSO control for 24 h.

Western blot. Cells were lysed in RIPA (50 mM Tris HCl, pH 7.4; 150 mM NaCl; 1 mM EDTA; 1% NP-40; 0.1% SDS; 0.5% sodium deoxycholate) buffer with 1X proteinase inhibitor from Roche (Mannheim, Germany) and incubated on ice for 20 minutes. Cell lysates were then transferred to new eppendorf tubes and vortexed for 1 minute. After vortexing, the lysates were centrifuged at 12,500 rpm for 10 minutes and the supernatants were collected. After centrifugation at 20,000 *g* for 10 min, the supernatants were collected. The quantity was determined with an infinite M200 (Tecan, Switzerland) using a BCA protein assay (Thermo Scientific Pierce™ BCA protein assay, Waltham, MA). All samples were diluted to equal protein concentrations by adding a proper volume of RIPA buffer. To break the protein structure, 6X sample buffer was added to each sample and mixed. The mixtures were heated at 95 ° C for 5 minutes. Denatured proteins were loaded in 6%~12% SDS–PAGE gels to separate the proteins with running buffer. A PVDF membrane from Millipore (Billerica, MA) was used to transfer protein samples from the gels to the membrane. The transfer system was used at 300 mA on ice for 2 hours. Membranes containing denatured proteins were blocked in TBST with 5% skim milk at room

temperature for 1 h. After blocking with milk, all membranes were washed 3 times for 10 minutes each with TBST. Then, all membranes were incubated with primary antibodies at 4 °C overnight. The membrane was washed in TBST and incubated with secondary antibodies in 5% skim milk for 1 h at room temperature. The membrane was washed in TBST again and then incubated with ECL from Millipore (Billerica, MA). The results were measured using a GE LAS-4000 (GE Healthcare Inc., Marlborough, MA). Antibodies used in the experiments are detailed in Table S8, Supporting Information. Uncropped films of the experiments displayed in the figures and Supplementary figures were showed in Figure S13, Supporting Information.

Quantitative RT–PCR. Quantitative RT–PCR (RT–qPCR) was performed using the StepOnePlus real-time PCR system (Applied Biosystems Inc., Foster City, CA). The primer sequences used for real-time PCR are listed in Table S8, Supporting Information.

Supplementary Figures

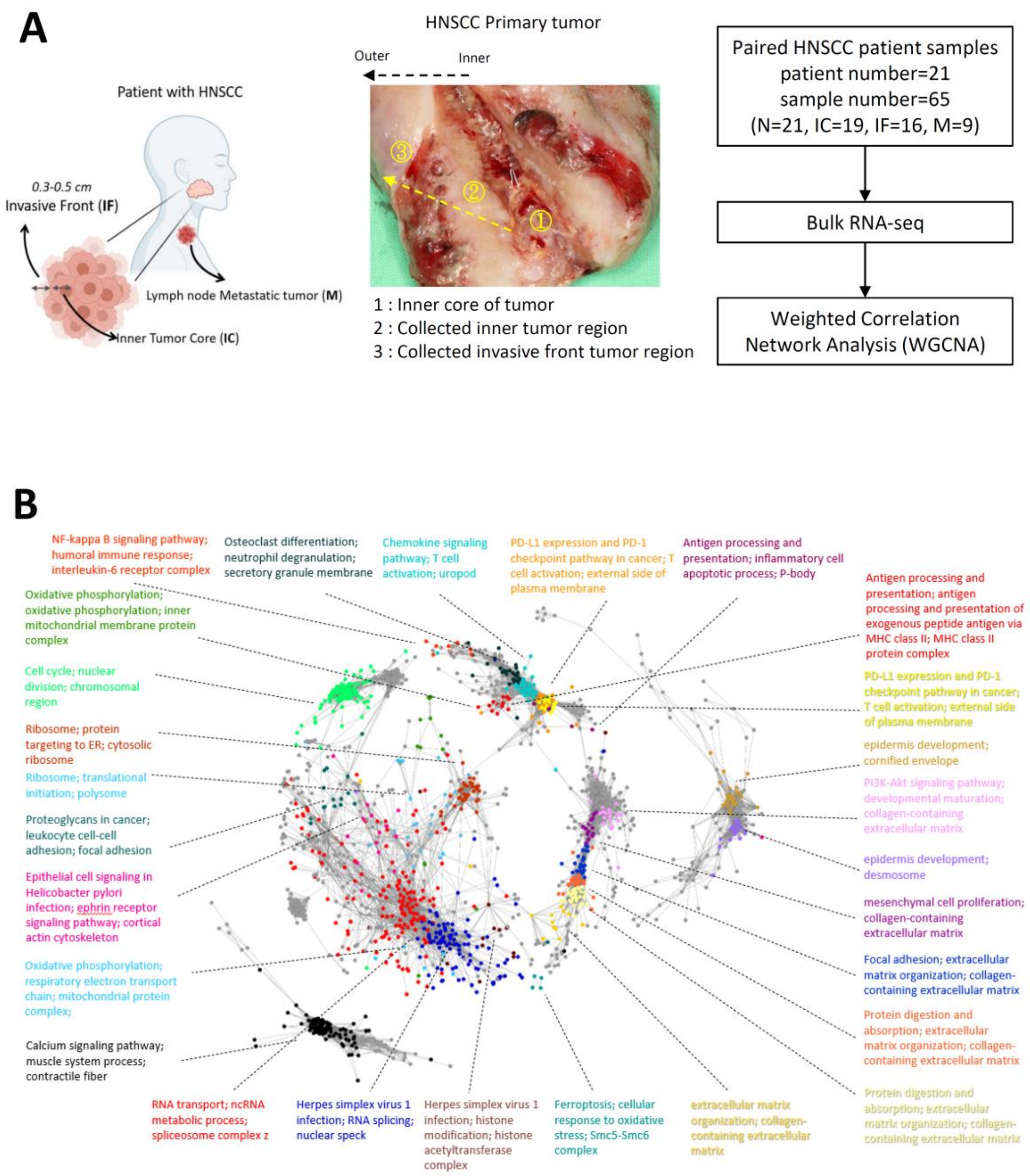

C

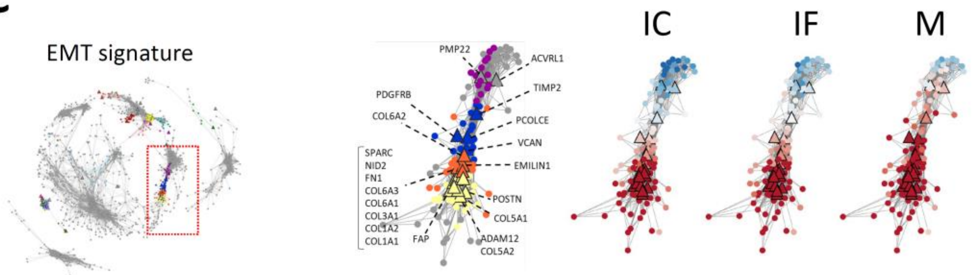

D

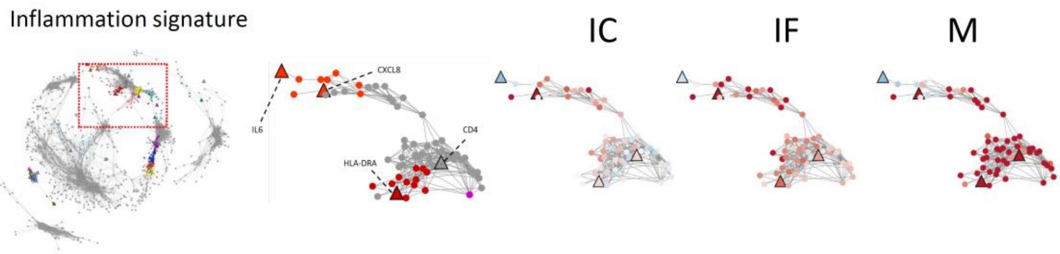

E

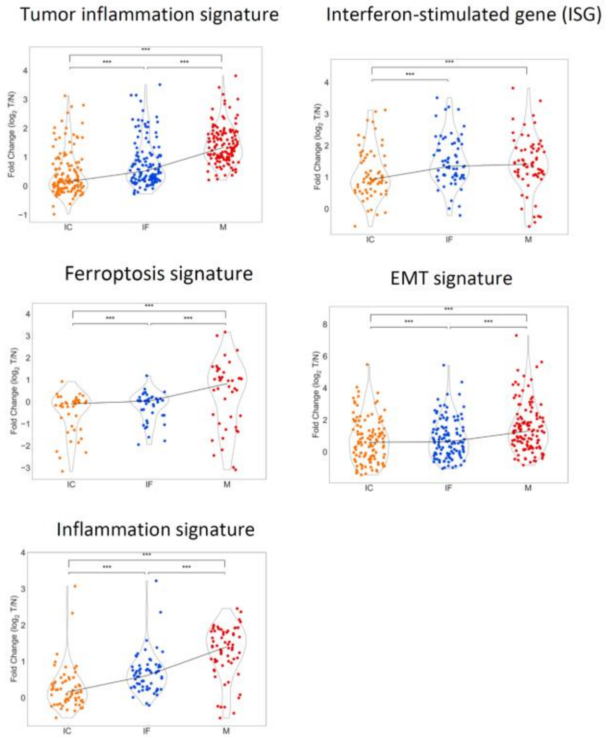

Signature	Site pair	p value (two-sided Wilcoxon signed-rank test)
EMT	IC-IF	3.77E-07
EMT	IF-M	3.36E-19
EMT	IC-M	1.17E-19
Ferroptosis	IC-IF	9.82E-07
Ferroptosis	IF-M	0.002234556
Ferroptosis	IC-M	5.51E-07
Inflammation	IC-IF	5.17E-12
Inflammation	IF-M	4.52E-07
Inflammation	IC-M	6.61E-10
ISG	IC-IF	1.64E-12
ISG	IF-M	0.342808794
ISG	IC-M	1.01E-06
TIS	IC-IF	2.09E-23
TIS	IF-M	6.78E-19
TIS	IC-M	3.70E-23

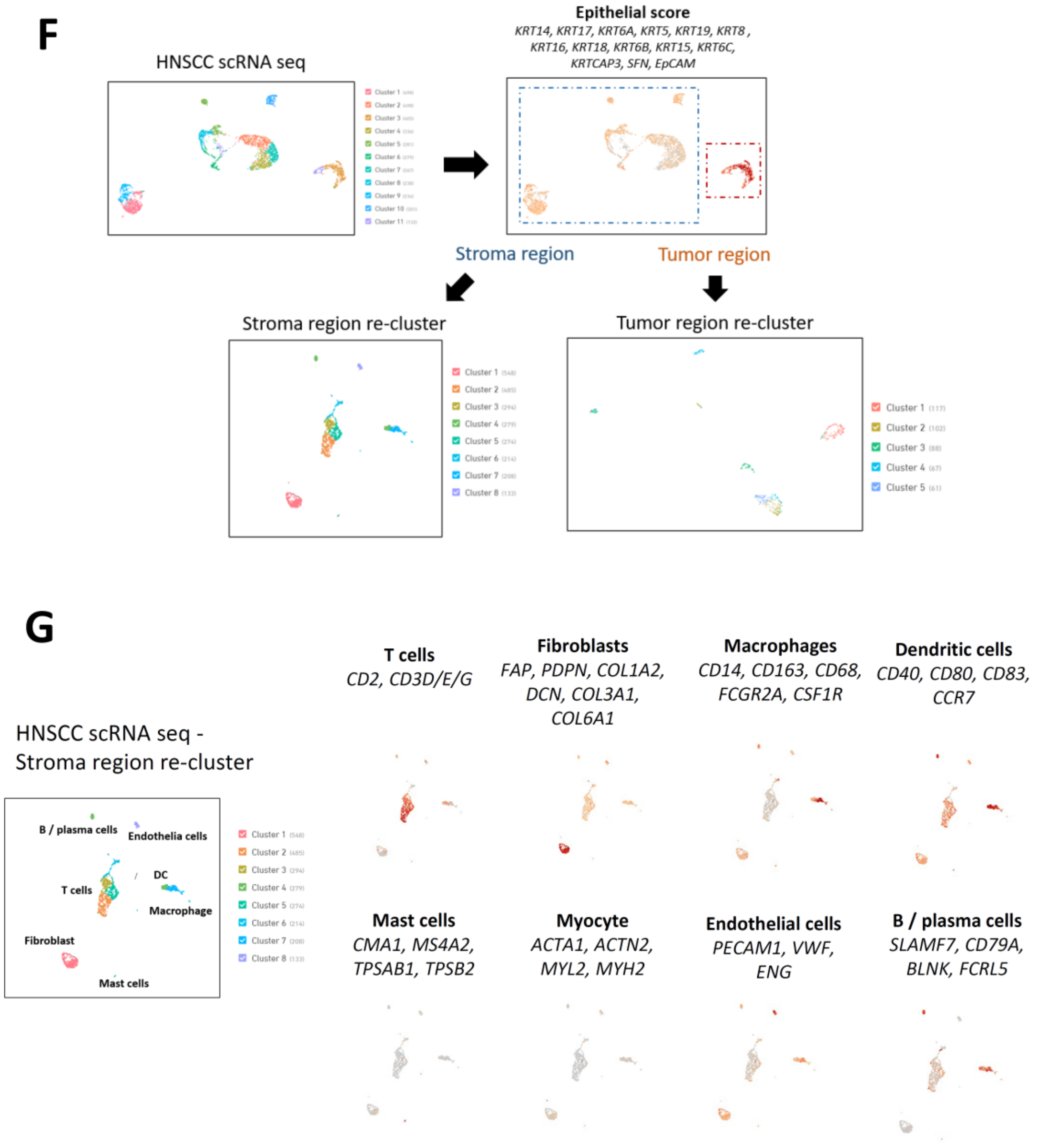

Supplementary Figure S1. **Transcriptomic analysis of different parts of HNSCC tumor samples.**

A, a schema for collecting the HNSCC samples from the inner tumor core (IC), invasive front (IF), and metastatic tumor (M) for bulk RNA sequencing (RNA-seq) and subsequent bioinformatics analysis. **B**, weighted correlation network analysis (WGCNA) of the 65 HNSCC samples. Gene correlation network with the modules characterized by significant enrichment for biological terms, including GO biological process (BP), GO cellular component (CC), and KEGG pathway annotations (hypergeometric test, Benjamini-Hochberg FDR q-value ≤ 0.05) are shown in different colors. **C**, overlay of the $\log_2$T/N values for each inner tumor core (IC), invasive front (IF), or metastatic (M) part on subnetworks of the network of B. Corresponding genes

(triangle) for EMT signature is shown for subnetworks. **D**, overlay of the $\log_2$T/N values for each inner tumor core (IC), invasive front (IF), or metastatic (M) part on subnetworks of the network of (B**)**. Corresponding genes (triangle) for inflammation signature is shown for subnetworks. **E**, Violin plots of fold changes (log2 T/N) for genes in the subnetworks comprising modules with enriched tumor inflammation, interferon-stimulated gene, ferroptosis, EMT, and inflammation signatures, of which HNSCC samples (T) derived from inner tumor core (IC), invasive front (IF), and metastatic tumor (M). The dots with the same color represent the log2 T/N values for the genes in the IC, IF, and M. The black line indicates the median of log2 T/N values among all genes in the IC, IF, and M. A triple asterisk indicates a p-value of <0.005 (two-sided Wilcoxon signed-rank test). **F**, schema of identification of tumor and non-tumor cell populations from scRNA-seq data by epithelial score (see reference 28). **G**, Mapping microenvironmental cell signatures in the stroma region of the scRNA-seq data.

A

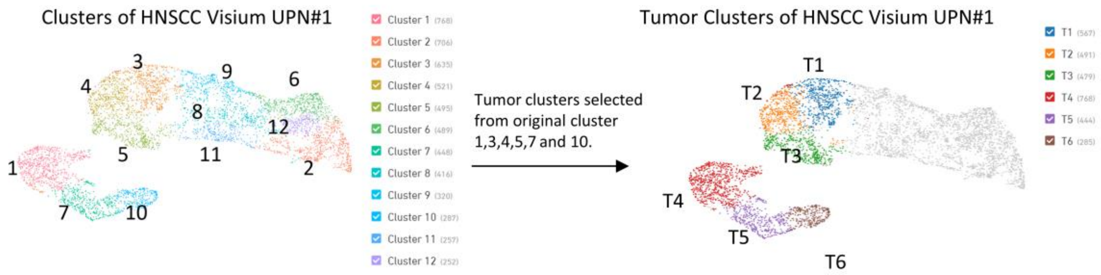

Clusters of HNSCC Visium UPN#1
Cluster 1
Cluster 2
Cluster 3
Cluster 4
Cluster 5
Cluster 6
Cluster 7
Cluster 8
Cluster 9
Cluster 10
Cluster 11
Cluster 12
Tumor clusters selected from original cluster 1,3,4,5,7 and 10.
Tumor Clusters of HNSCC Visium UPN#1
T1
T2
T3
T4
T5
T6

B

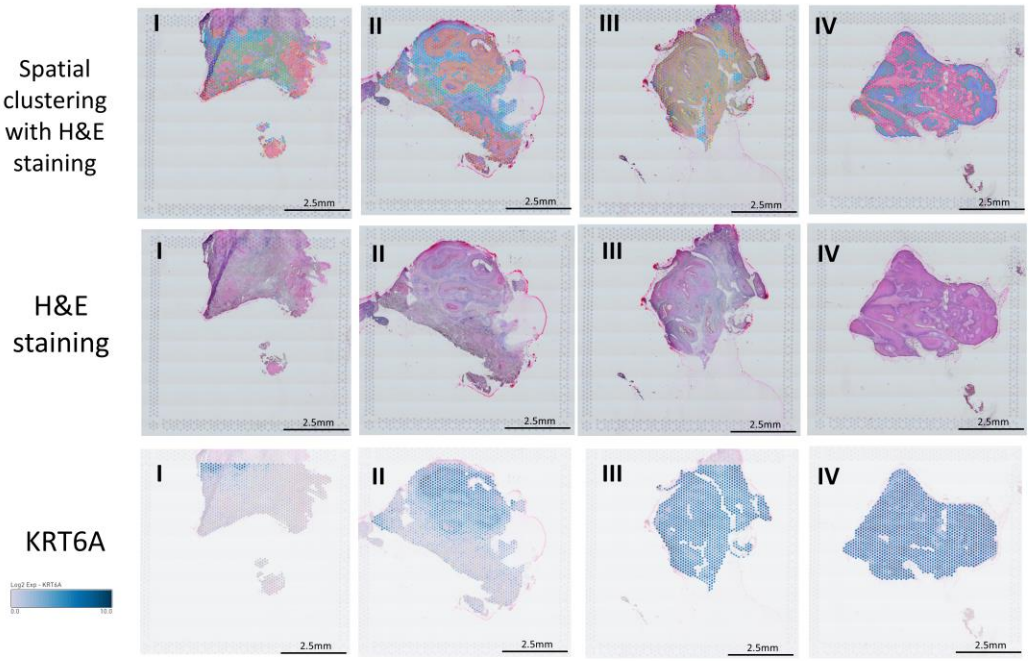

Spatial clustering with H&E staining
H&E staining
KRT6A
I
II
III
IV
2.5mm

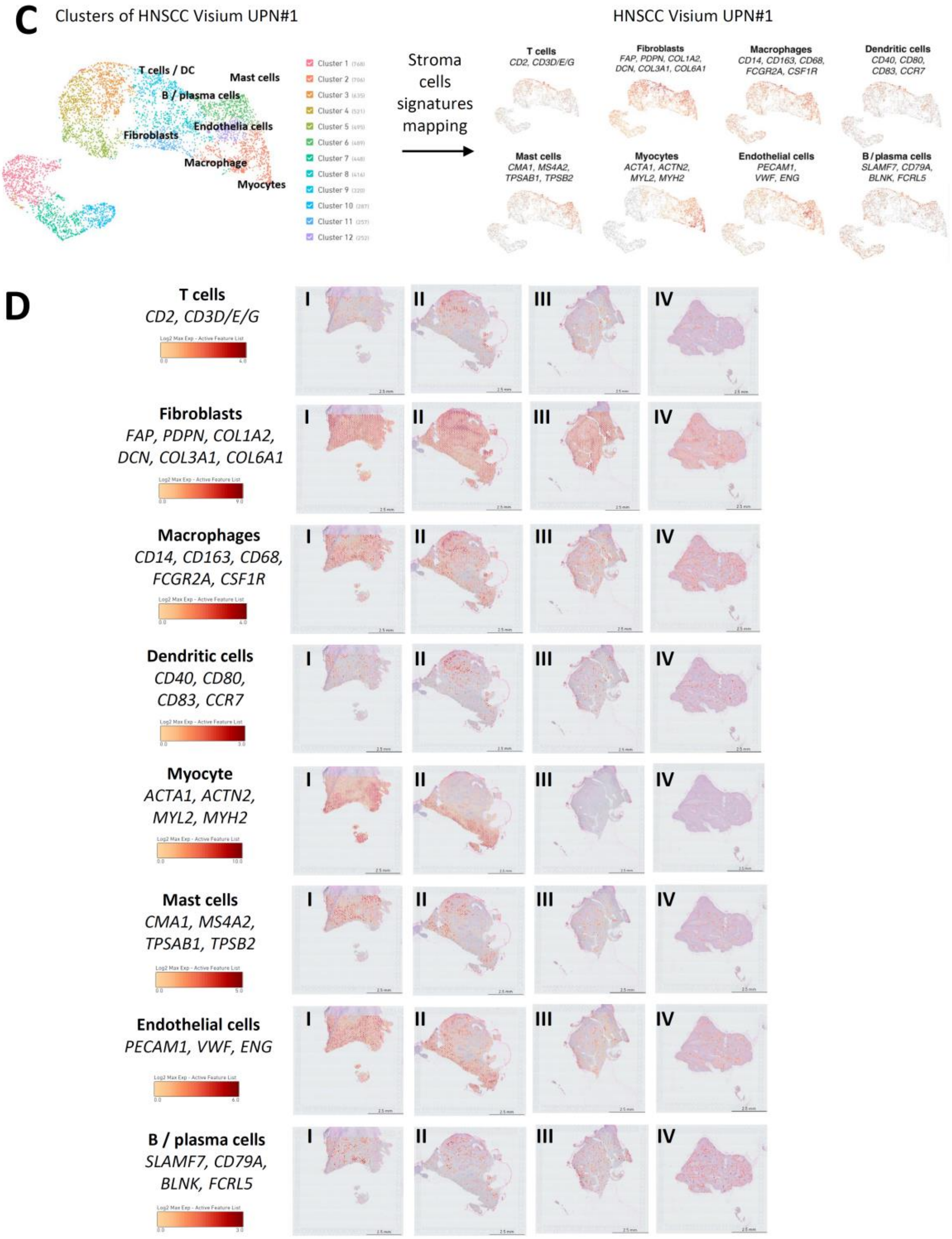

Supplementary Figure S2. The spatial correlation between EMT and ferroptosis signature in HNSCC sample No. Visium-1.

A, UMAP for showing the 12 clusters of the Visium spatial transcriptomic analysis from samples of No. Visium-1 HNSCC case and selected 6 tumor clusters were showed in right. **B**, upper, the spatial clustering distribution of the clusters from Visium analysis from No. Visium-1 HNSCC sample. Middle, the H&E staining of the four serial

sections from No. Visium-1 HNSCC sample. Lower, the KRT6A expression of the fours serial section from No. Visium-1 HNSCC sample. **C**, UMAP for mapping microenvironmental cell signatures in HNSCC sample No. Visium-1. **D**, mapping microenvironmental cell signatures in the four serial sections from No. Visium-1 HNSCC sample.

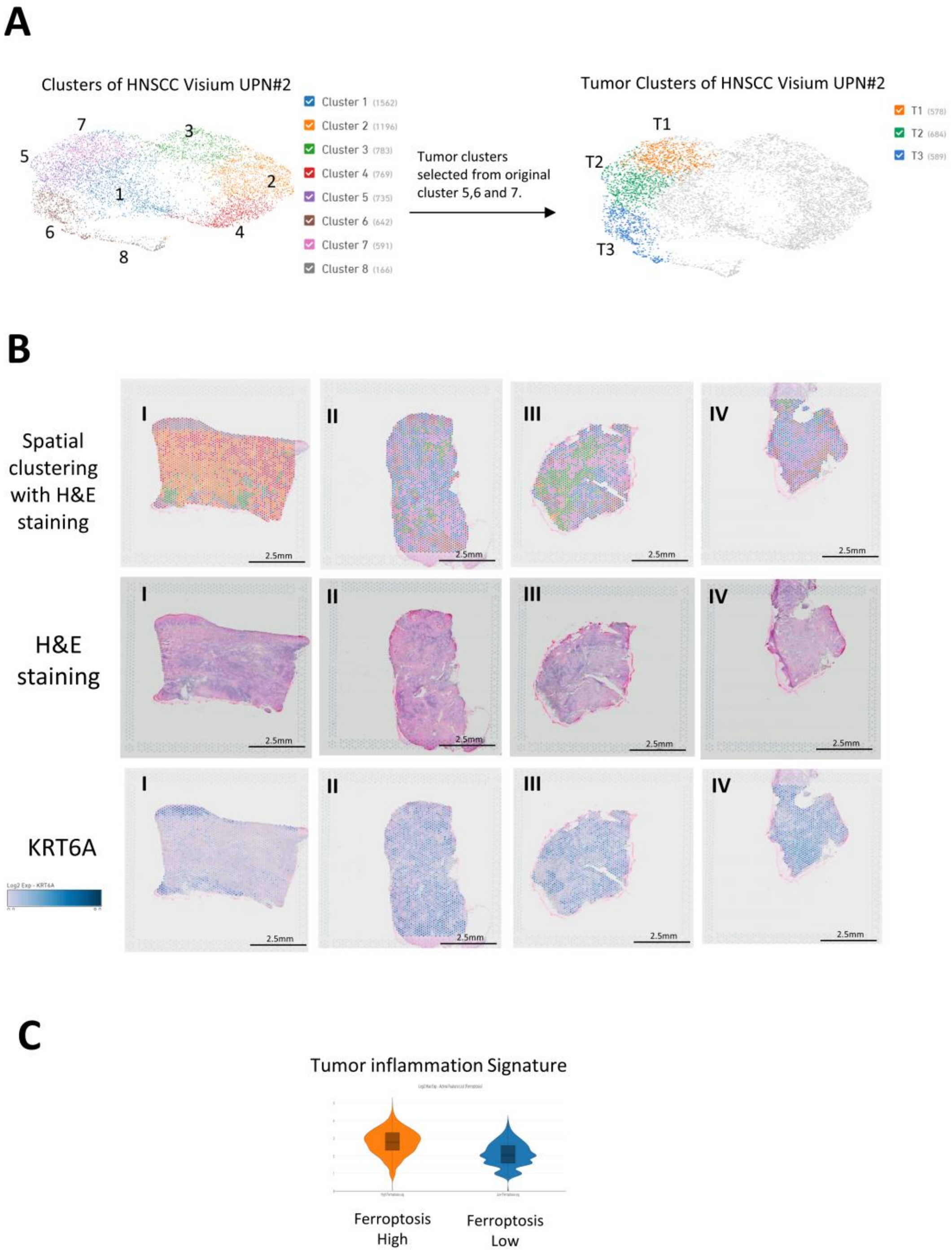
A
Clusters of HNSCC Visium UPN#2
Cluster 1 (1562)
Cluster 2 (1196)
Cluster 3 (783)
Cluster 4 (769)
Cluster 5 (735)
Cluster 6 (642)
Cluster 7 (591)
Cluster 8 (166)
Tumor clusters selected from original cluster 5,6 and 7.
Tumor Clusters of HNSCC Visium UPN#2
T1 (578)
T2 (684)
T3 (589)
B
Spatial clustering with H&E staining
H&E staining
KRT6A
I
II
III
IV
2.5mm
C
Tumor inflammation Signature
Ferroptosis High
Ferroptosis Low

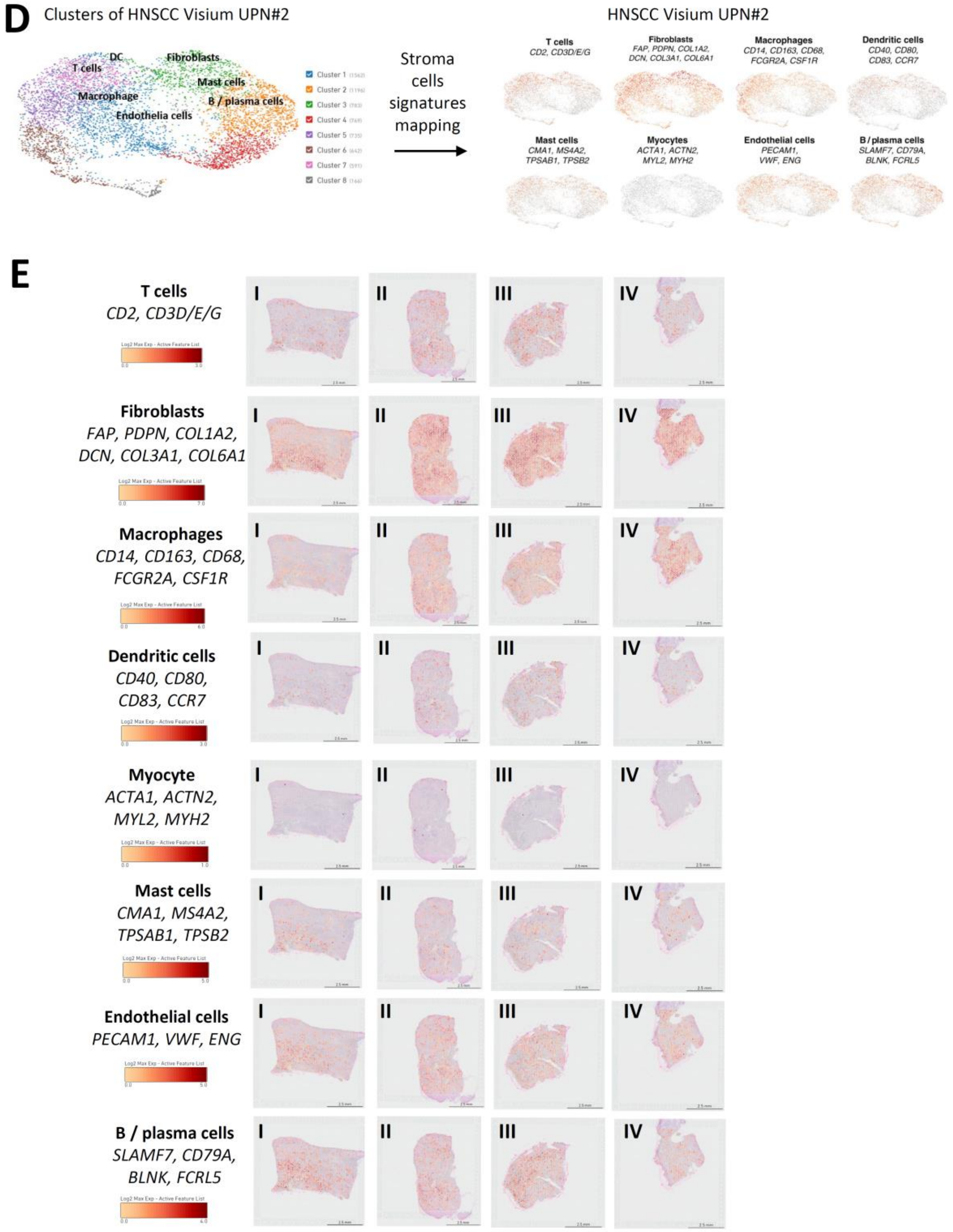

Supplementary Figure S3. The spatial correlation between EMT and ferroptosis signature in HNSCC sample No. Visium-2.

A, UMAP for showing the 8 clusters of the Visium spatial transcriptomics analysis from samples of No. Visium-2 case and selected 3 tumor clusters were showed in right. **B**, upper, the spatial clustering distribution of the clusters from Visium analysis from No.

Visium-2 HNSCC sample. Middle, the H&E staining of the four serial sections from No. Visium-2 HNSCC sample. Lower, the KRT6A expression of the fours serial section from No. Visium-2 HNSCC sample. **C**, violin plots for showing the expression of tumor inflammation signature in ferroptosis high and low group of No. Visium-2 HNSCC sample in Visium analysis. **D**, UMAP for mapping microenvironmental cell signatures in HNSCC sample No. Visium-2. **E**, mapping microenvironmental cell signatures in the four serial sections from No. Visium-2 HNSCC sample.

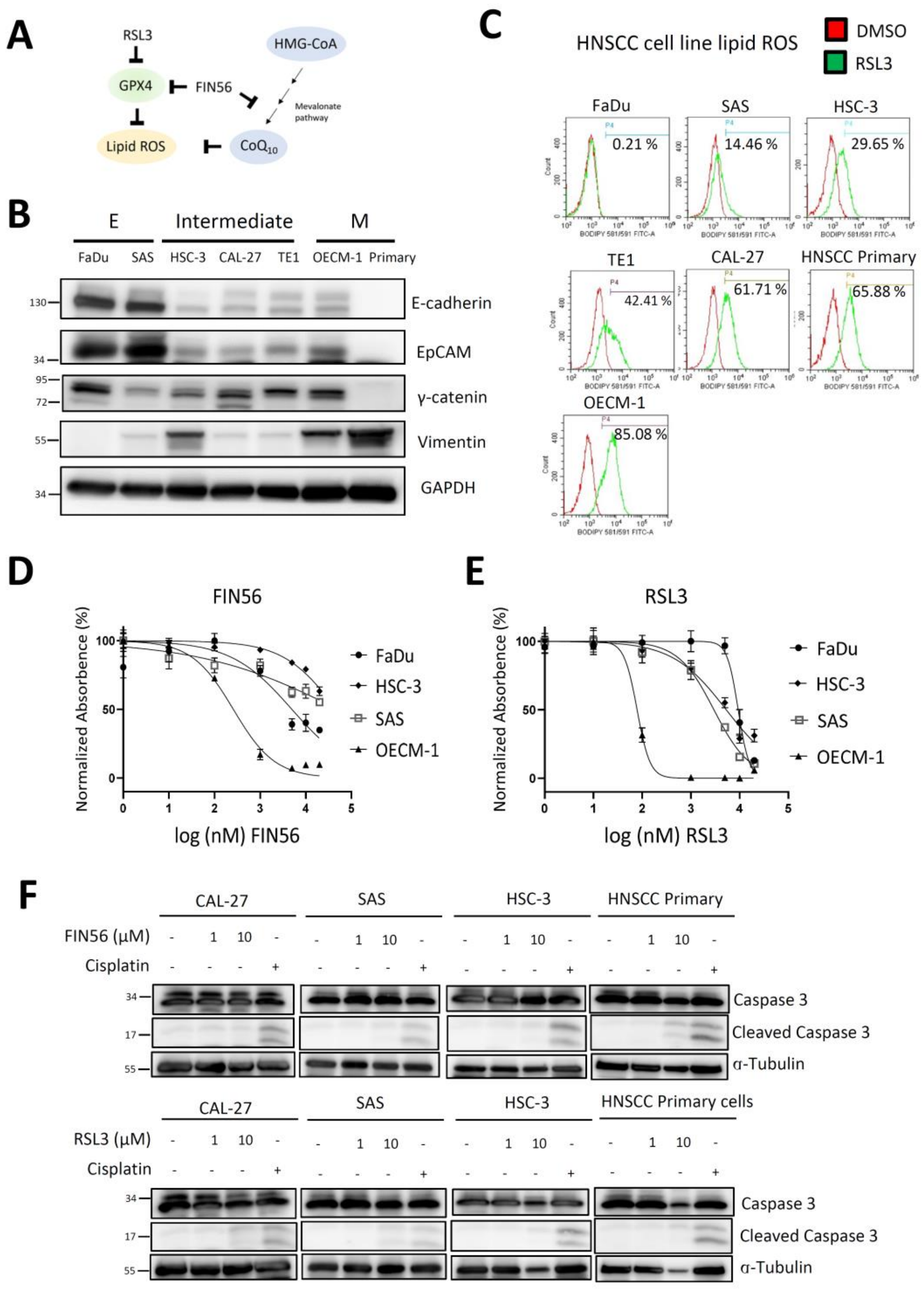

Supplementary Figure S4. Examination of the EMT feature and susceptibility to ferroptosis inducers in different HNSCC cell lines.

A, schema for illustrating the pathways inhibited by RSL3 and FIN56. **B**, western blots of the epithelial markers (E-cadherin, EpCAM, γ-catenin) and mesenchymal marker (vimentin) of 6 HNSCC cell lines and a primary HNSCC culture. GAPDH was a loading control. **C**, flow cytometry for detecting the lipid ROS of six HNSCC cell lines

(FaDu, HSC-3, SAS, TE1, CAL-27, OECM-1) and a primary HNSCC culture treated with RSL3 1μM for 4h or a control vehicle DMSO. **D**, viability assay of four HNSCC cells (FaDu, HSC-3, SAS, OECM-1) treated with different doses of FIN56. n=5 for each dose. **E**, viability assay of four HNSCC cells (FaDu, HSC-3, SAS, OECM-1) treated with different doses of RSL3. n=5 for each dose. **F**, western blots of the cleaved caspase-3 and non-cleaved caspase-3 in HNSCC cell treated with FIN56 1μM and 10μM and RSL3 1μM and 10μM for 24h. HNSCC cell treated with cisplatin 50μM for 24h was a positive control.

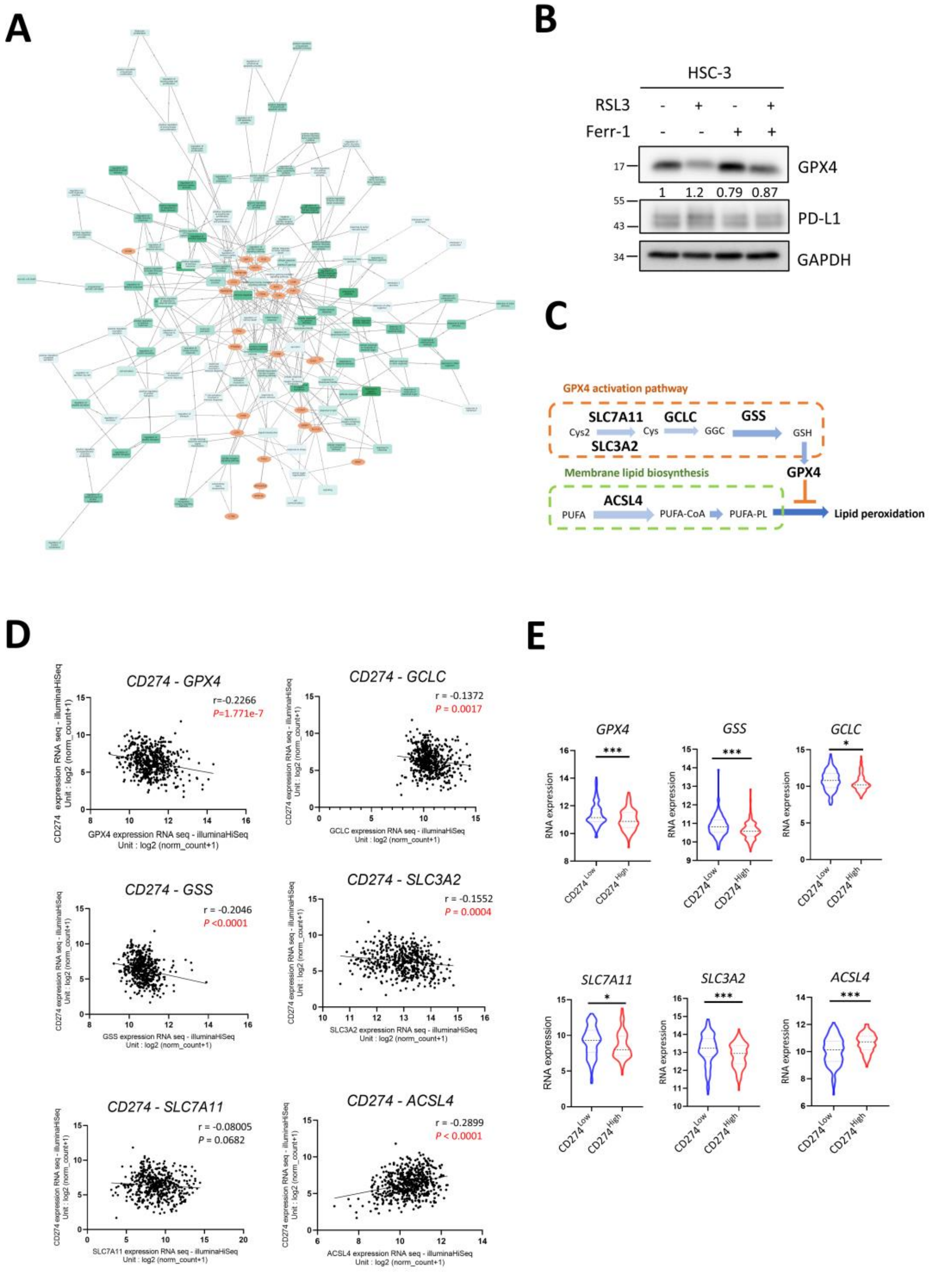

Supplementary Figure S5. Ferroptosis stress induces PD-L1 expression.

A, the result of GO analysis of the 42 overlapping genes regulated by ferroptosis. The zoom-in picture is shown in Figure 3c. **B**, western blots of GPX4 and PD-L1 of HSC-3 cells treated with RSL3 1 μM for 48 h or a vehicle control with/without co-treatment with the ferroptosis inhibitor ferri-1 2 μM for 48 h. GAPDH was a loading control. **C**, a schema for illustrating the genes related to GPX4 activation pathway and membrane lipid biosynthesis. **D**, correlation between the expression of *CD274-GPX4, CD274-GSS,*

CD274-GCLC, CD274-SLC7A11, CD274-SLC3A2 and *CD274-ACSL4* in the TCGA HNSCC samples (n=520). Pearson's correlation coefficient and the corresponding p-value are shown. **E**, relative expression of *GPX4*, *GSS*, *GCLC*, *SLC7A11*, *SLC3A2*, and *ACSL4* in CD274_high vs. CD274_low group of the HNSCC samples from TCGA dataset. CD274_low group n=130 and CD274_high group n=129 *p<0.05, ***p<0.001 (Student's t test).

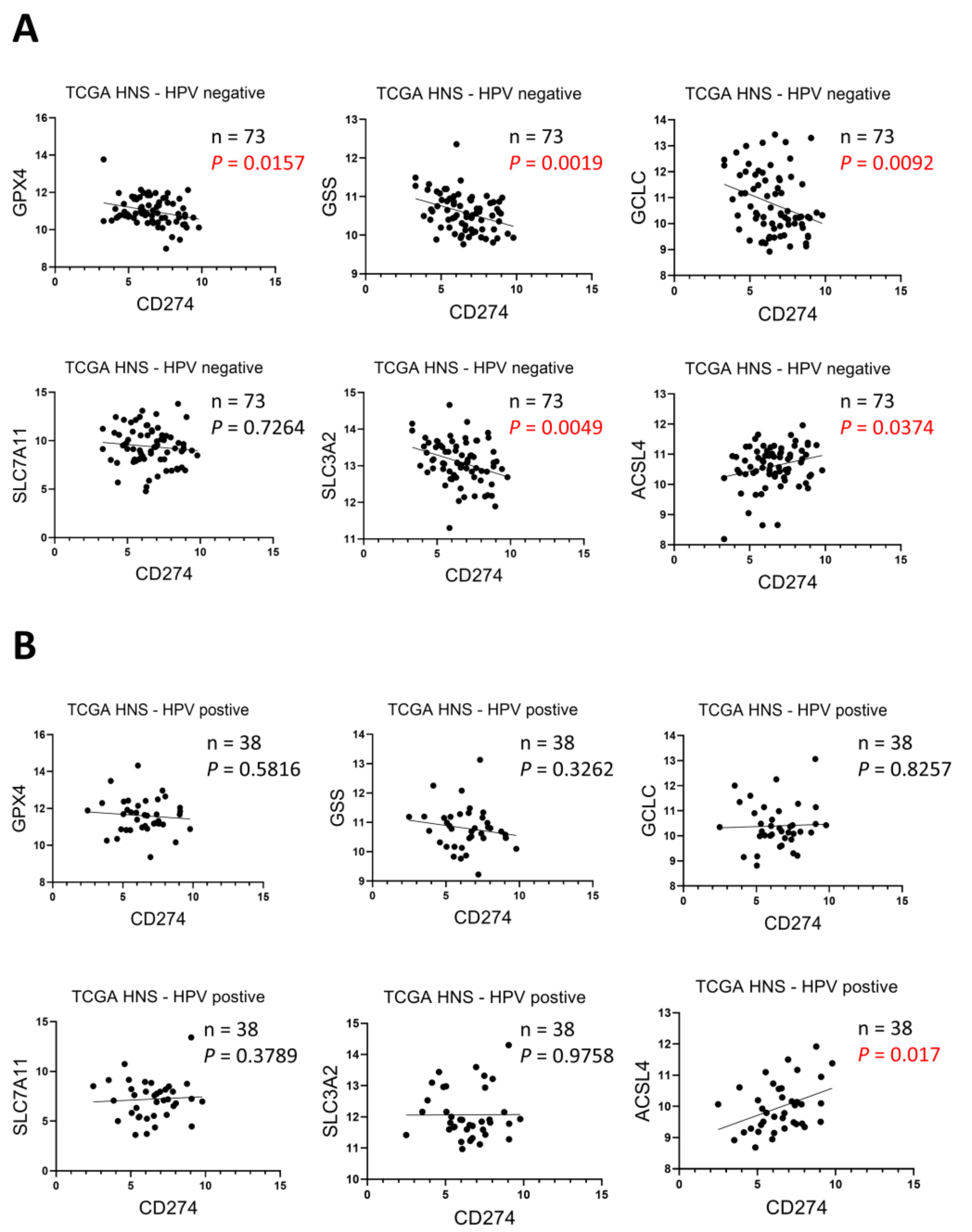

Supplementary Figure S6. Correlation of PD-L1 and GPX4 expression in the HPV-negative and HPV-positive HNSCC sample from TCGA-HNSCC dataset.
A, correlation between the expressions of *CD274-GPX4, CD274-GSS, CD274-GCLC, CD274-SLC7A11, CD274-SLC3A2* and *CD274-ACSL4* in TCGA HNSCC HPV-negative samples (n=73). Pearson's correlation coefficient and the corresponding p-value are shown. **B**, correlation between the expressions of *CD274-GPX4, CD274-GSS, CD274-GCLC, CD274-SLC7A11, CD274-SLC3A2* and *CD274-ACSL4* in TCGA HNSCC HPV-positive samples (n=38). Pearson's correlation coefficient and the corresponding p-value are shown.

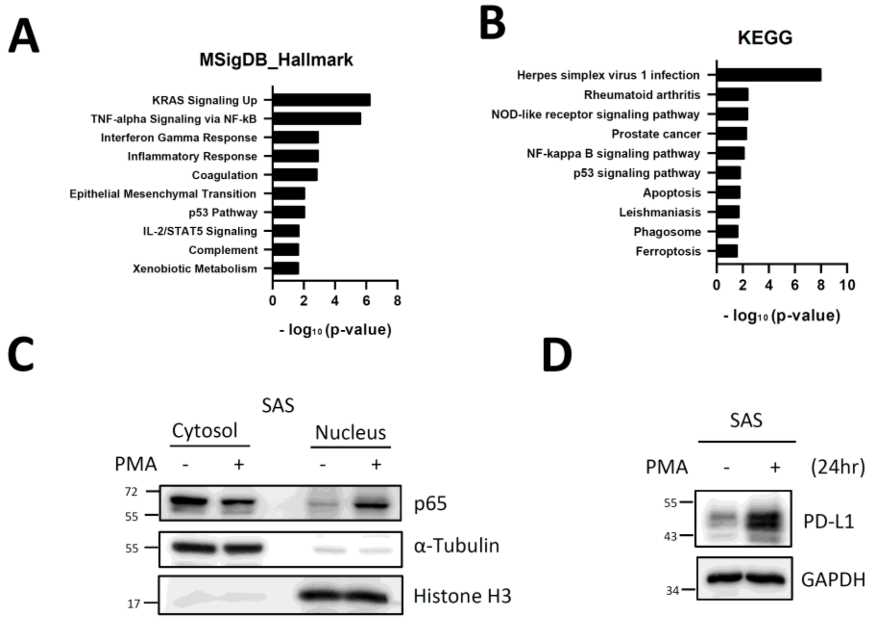

Supplementary Figure S7. NF-κB and calcium signaling activate the expression of PD-L1 in HNSCC.

A, Molecular Signatures Database (MSigDB) hallmark analysis for identifying the 539 up-regulation genes of RNA seq data from HSC-3 cells treated with FIN56 1μM for 48 h compare with DMSO control. **B**, KEGG analysis for identifying the 539 up-regulation genes of RNA seq data from HSC-3 cells treated with FIN56 1μM for 48 h compare with DMSO control. **C**, western blots of PD-L1 SAS cells treated with PMA 10ng/ml for 24 h. **D**, nuclear-cytoplasmic fractionation, and western blots of p65 of SAS cells treated with PMA 10ng/ml for 24 h for activation of NF-κB. α-tubulin was a control of cytoplasmic protein, and histone H3 was a control of nuclear protein.

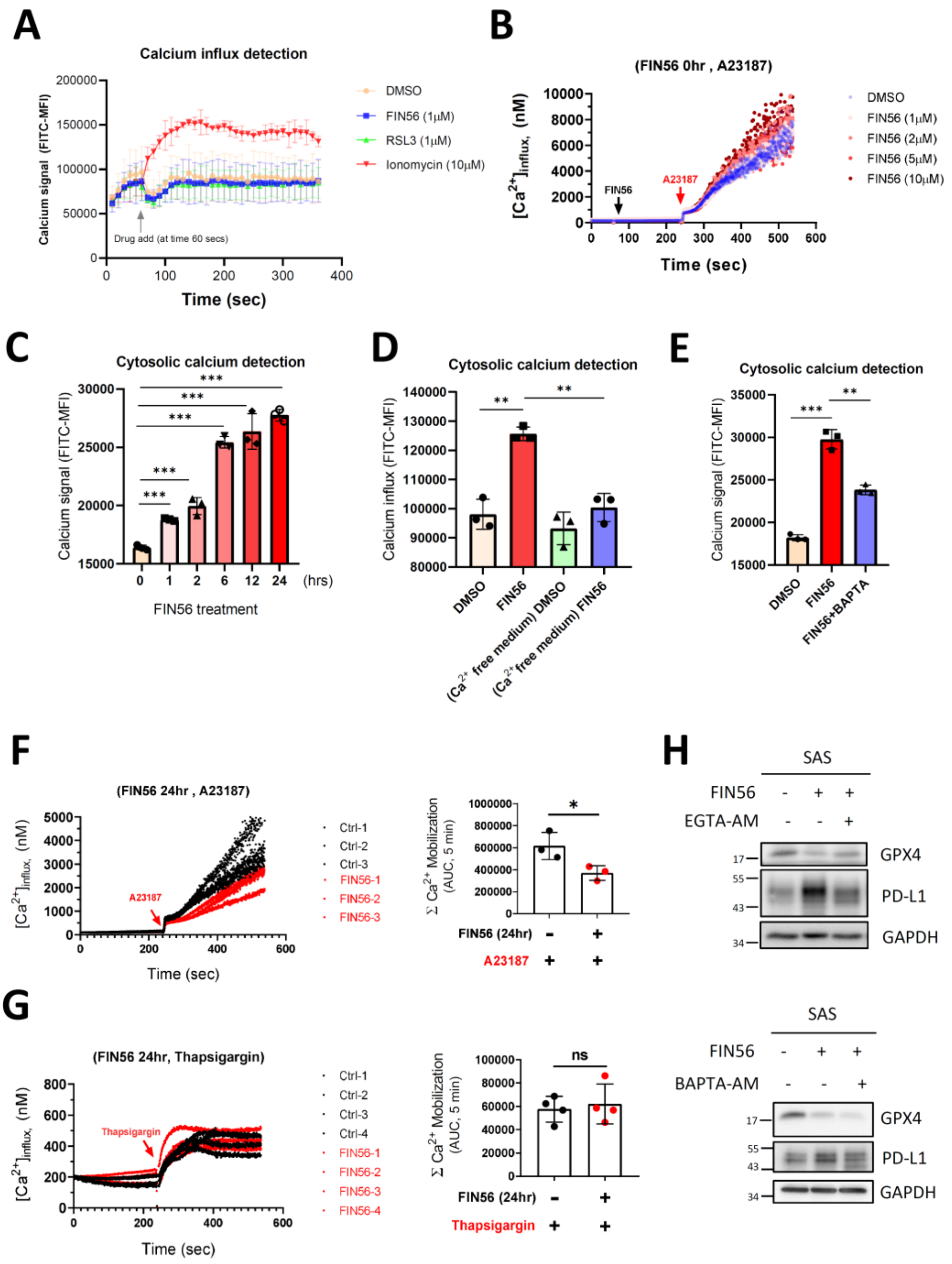

Supplementary Figure S8. Ferroptosis induced calcium signaling.

A, Fluo-4-labeled CAL-27 cells treated with DMSO, FIN56 1μM, RSL3 1μM or Ionomycin 10μM for 5 mins intracellular calcium signal detection. Calcium FITC-MFI detection for every 10 sec. The data is presented in mean ± S.D, n=3. **B**, Fluo-4-labeled CAL-27 cells were treated with indicated concentrations of FIN56 (0, 1, 2, 5, 10μM) for 3 min before being challenged with calcium ionophore A23187 5μM. n=1 for each

experiment. **C**, CAL-27 cells were treated with FIN56 1μM for indicated time (0, 1, 2 ,6 ,12 ,24 h) for Fluo-4-labeled and detection of calcium signal. The data is presented in mean ± S.D, n=3. ***p<0.001 (Student's t test). **D**, CAL-27 cells were treated with FIN56 1μM or DMSO control in calcium containing or free medium for 6 h for Fluo-4-labeled and detection of calcium signal. The data is presented in mean ± S.D, n=3. **p<0.01 (Student's t test). **E**, CAL-27 cells were treated with FIN56 1μM, BAPTA-AM 10μM or DMSO control for 24 h for Fluo-4-labeled and detection of calcium signal. The data is presented in mean ± S.D, n=3. **p<0.01, ***p<0.001 (Student's t test). **F**, left, CAL-27 cells were treated with FIN56 1μM or DMSO control for 24 h for Fluo-4-labeled and challenged with calcium ionophore A23187 5μM for calcium mobilization detection. Right, a histogram for presenting the quantified result of calcium mobilization. The data is presented in mean ± S.D, n=3. *p<0.05 (Student's t test). **G**, left, CAL-27 cells were treated with FIN56 1μM or DMSO control for 24 h for Fluo-4-labeled and challenged with thapsigargin 4μM for calcium mobilization detection. Right, a histogram for presenting the quantified result of calcium mobilization. The data is presented in mean ± S.D, n=4. (Student's t test). **H**, up, western blots of GPX4 and PD-L1 in SAS cells treated with FIN56 1μM for 24 h or a vehicle control with/without co-treatment with the calcium chelator EGTA-AM 10μM for 24 h. Down, western blots of GPX4 and PD-L1 in SAS cells treated with FIN56 1μM for 24 h or a vehicle control with/without co-treatment with the calcium chelator BAPTA-AM 10μM for 24 h. GAPDH was a loading control.

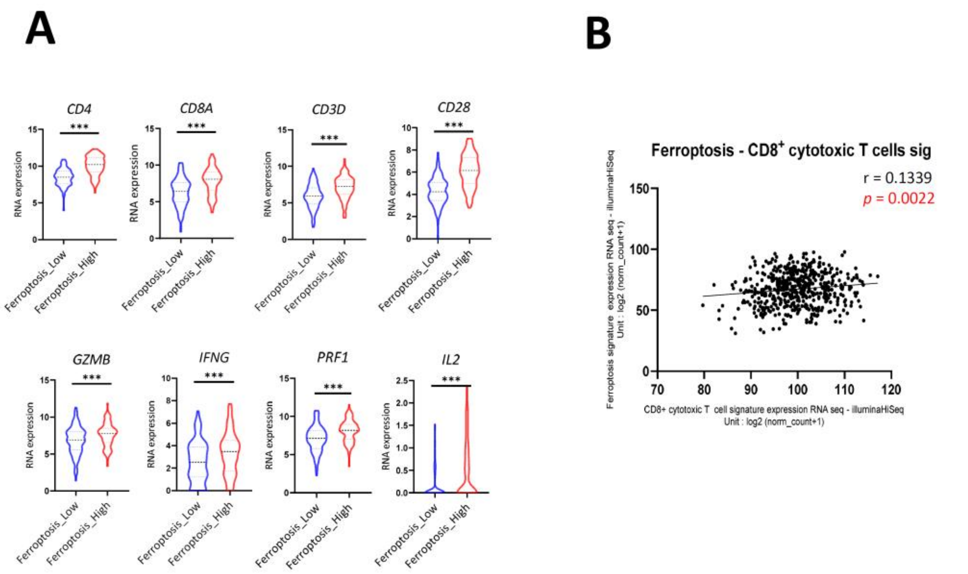

C

Correlation of *GPX4* expression and T cell infiltration in TCGA HNSC (by TIMER 2.0)

Spearman's ρ : positive correlation (p<0.05, ρ>0)
Spearman's ρ : negative correlation (p<0.05, ρ<0)
Spearman's ρ : not significant (p>0.05)

cancer	T cell CD8+ TIMER	T cell CD8+ EPIC	T cell CD8+ MCP-COUNTER	T cell CD8+ CIBERSORT	T cell CD8+ CIBERSORT-ABS	T cell CD8+ QUANTISEQ	T cell CD8+ XCELL	T cell CD8+ naive XCELL	T cell CD8+ central memory XCELL	T cell CD8+ effector memory XCELL
HNSC (n=522)	-0.402	-0.198	0.146	0.027	0.13	0.083	-0.046	-0.077	0.026	0.042
HNSC-HPV- (n=422)	-0.394	-0.37	0.042	-0.095	0.022	-0.023	-0.195	-0.159	-0.112	-0.035
HNSC-HPV+ (n=98)	-0.283	0.392	0.445	0.381	0.418	0.396	0.414	0.309	0.416	0.325

D

T cell infiltration score in TCGA HNSC (by CIBERSORT)

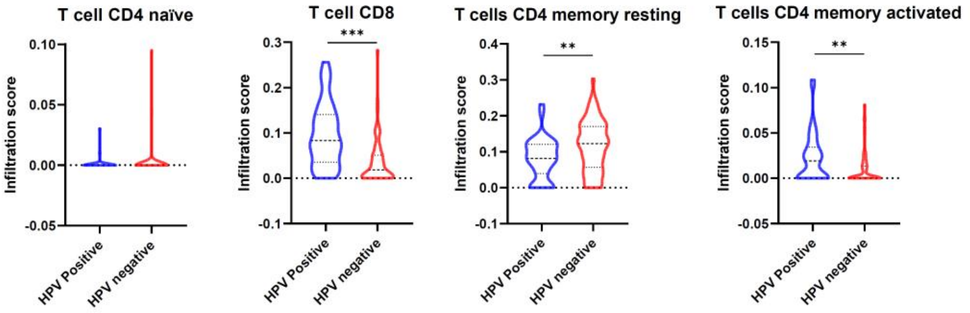

E

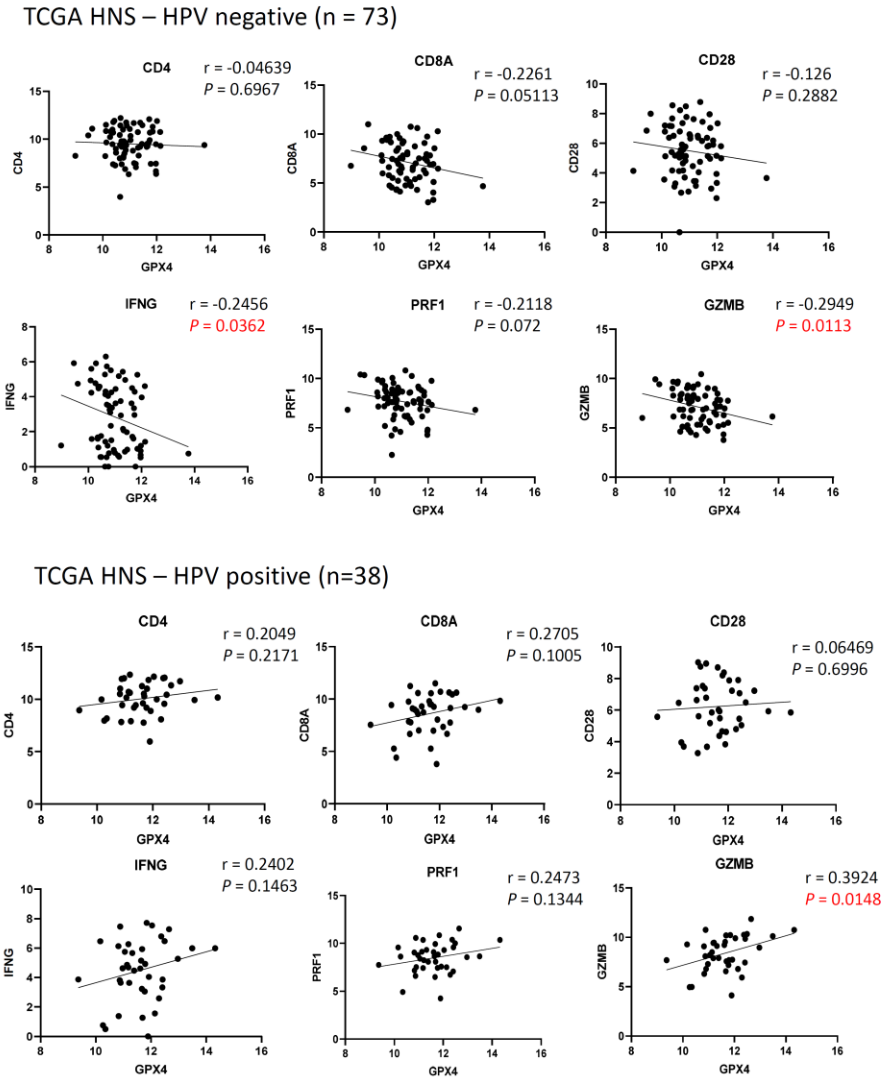

Supplementary Figure S9. Correlation of *GPX4* and T cell activation signature of HPV-positive and negative cases from TCGA-HNSCC dataset.

A, relative expression of the immune-related genes CD274, CD4, CD8A, CD3D, CD28, GZMB, IFNG, PRF1, and IL2 in the ferroptosis_high vs. ferroptosis_low group of patients with HNSCC from TCGA-HNSCC database. Ferroptosis _low group n=130 and ferroptosis _high group n=129 **p*<0.05, ****p*<0.001 (Student's t test). **B**, correlation between the expression of ferroptosis signature and $CD8^+$ cytotoxic T cells signature in TCGA HNSCC dataset (n=520). Pearson's correlation coefficient and the corresponding p-value are shown. **C**, a functional heatmap table shows the association between GPX4 expression and immune infiltration level of multiple $CD8^+$ T cell types of TCGA-HNSCC dataset by TIMER 2.0. **D**, analysis of T cell infiltration score of TCGA-HNSCC HPV-positive sample (n=38) and negative sample (n=73) by

CIBERSORT. **p<0.01, ***p<0.001 (Student's t test). **E**, correlation between the expression of *GPX4* and activated T cell-related genes expression (*CD4, CD8a, CD28, IFNG, PRF1* and *GZMB*) in TCGA-HNSCC HPV-positive sample (n=38) and negative sample (n=73). Pearson's correlation coefficient and the corresponding p-value are shown.

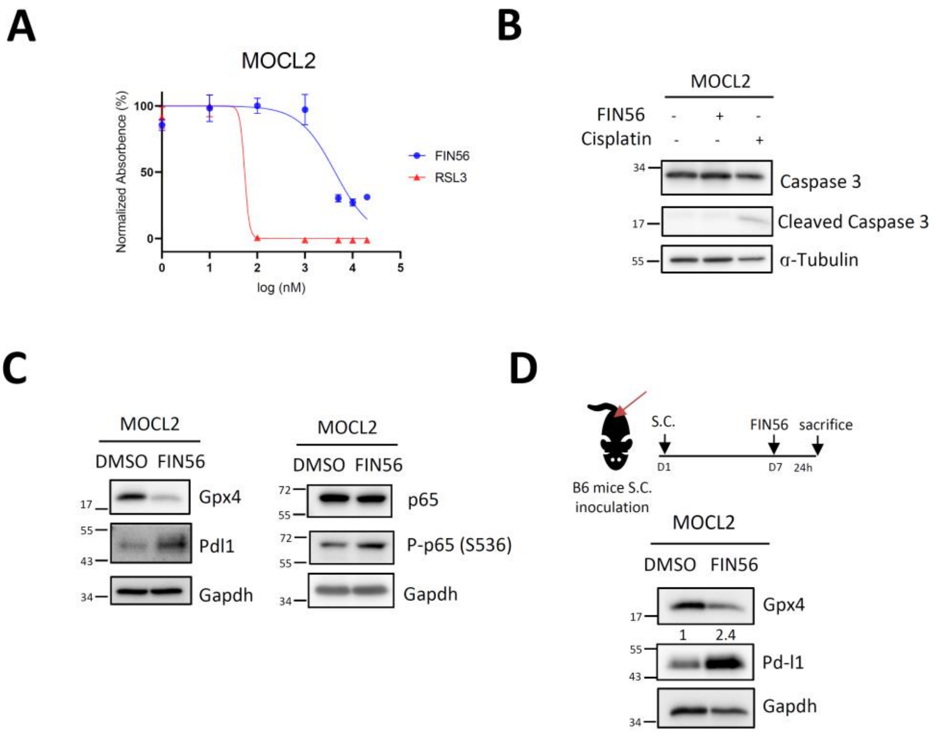

Supplementary Figure S10. Ferroptosis induces PD-L1 expression in murine HNSCC cells.

A, cell viability assay of the murine HNSCC cell line MOCL2 treated with FIN56 and RSL3 with the indicated concentrations for 24 h. **B**, western blots of the cleaved and non-cleaved caspase 3 of MOCL2 cells treated with FIN56 1 μM or DMSO for 24 h. GAPDH was a loading control. MOCL2 cell treated with cisplatin 50μM for 24 h was a positive control treatment. **C**, left, western blots of the total and serine 536-phosphorylated p65 of MOCL2 cells treated with FIN56 1μM or DMSO for 24h. GAPDH was a loading control. Right, western blots of GPX4 and PD-L1 of MOCL2 cells treated with FIN56 1μM or DMSO for 24h. GAPDH was a loading control. **D**, upper, schema for the syngeneic murine HNSCC experiment. 1x107 of the murine HNSCC cell line MOCL2 cells were inoculated in the subcutaneous region of C57BL/6J mice. FIN56 (100mg/kg) were intratumorally injected 7 days after tumor inoculation. Mice were sacrificed after 24 h. The tumors were harvested for experiments. Lower, western blot of GPX4 and PD-L1 of MOCL2-formed syngeneic tumor treated with FIN56 or a vehicle control. GAPDH was a loading control.

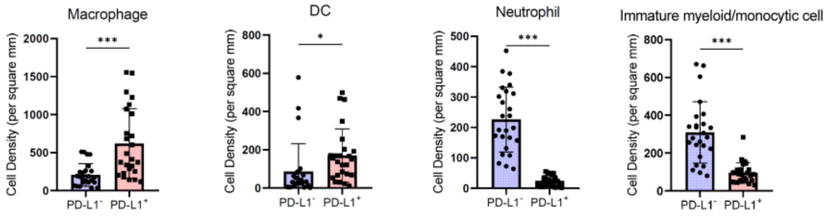

Supplementary Figure S11. Multispectral immunofluorescent imaging of HNSCC patient slide to identify PD-L1 expression in immune cell.

Cell density of PD-L1$^-$ or PD-L1$^+$ cells in macrophage, DC, neutrophil or immature myeloid/monocytic cell of multiplex immunostaining analysis. The data is presented in mean ± S.D, n=25. *p<0.05, ***p<0.001 (Student's t test).

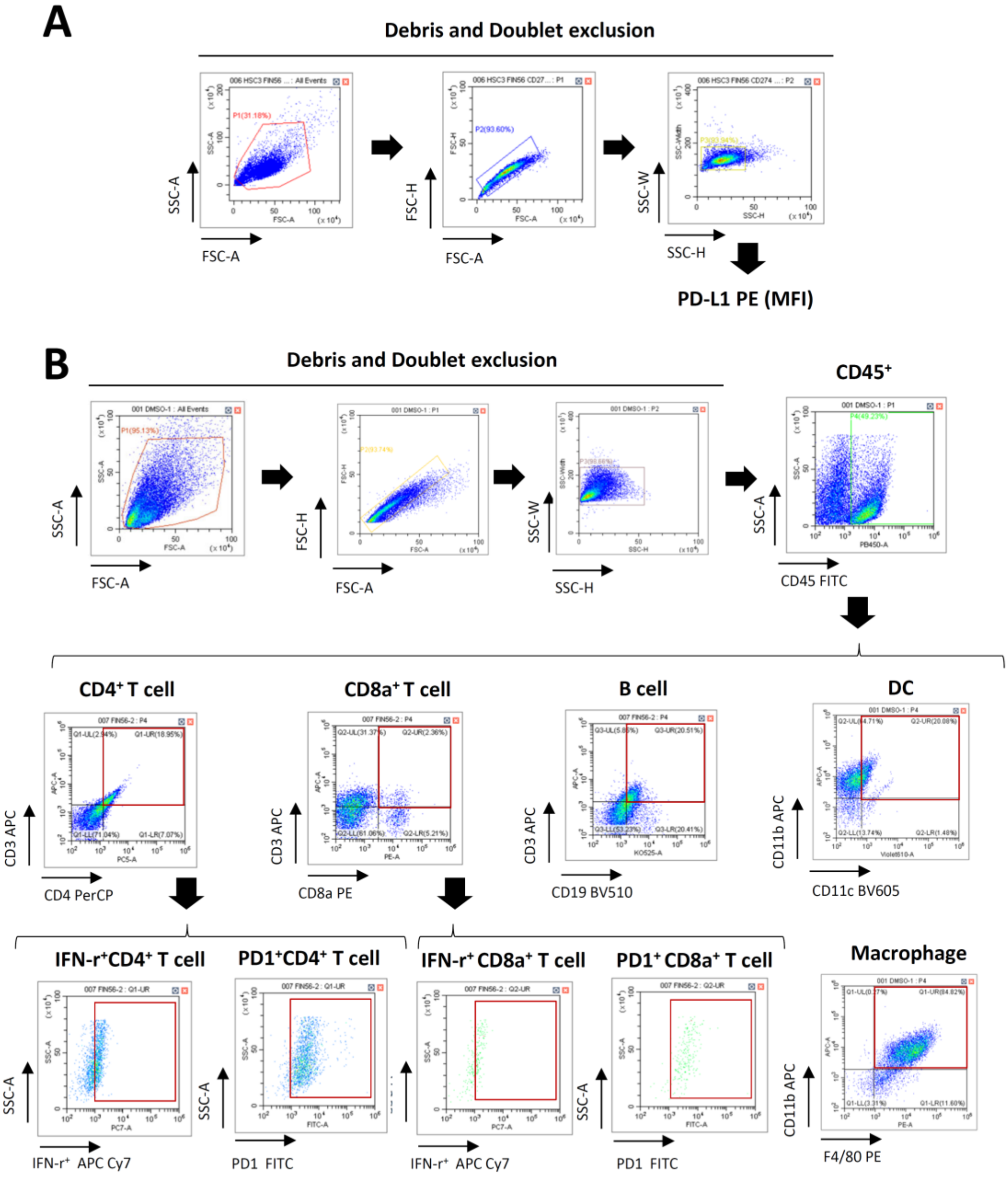
A
Debris and Doublet exclusion
SSC-A
FSC-A
FSC-H
FSC-A
SSC-W
SSC-H
PD-L1 PE (MFI)
B
Debris and Doublet exclusion
CD45+
SSC-A
FSC-A
FSC-H
FSC-A
SSC-W
SSC-H
SSC-A
CD45 FITC
CD4+ T cell
CD8a+ T cell
B cell
DC
CD3 APC
CD4 PerCP
CD3 APC
CD8a PE
CD3 APC
CD19 BV510
CD11b APC
CD11c BV605
IFN-r+CD4+ T cell
PD1+CD4+ T cell
IFN-r+ CD8a+ T cell
PD1+ CD8a+ T cell
Macrophage
SSC-A
IFN-r+ APC Cy7
SSC-A
PD1 FITC
IFN-r+ APC Cy7
SSC-A
PD1 FITC
CD11b APC
F4/80 PE

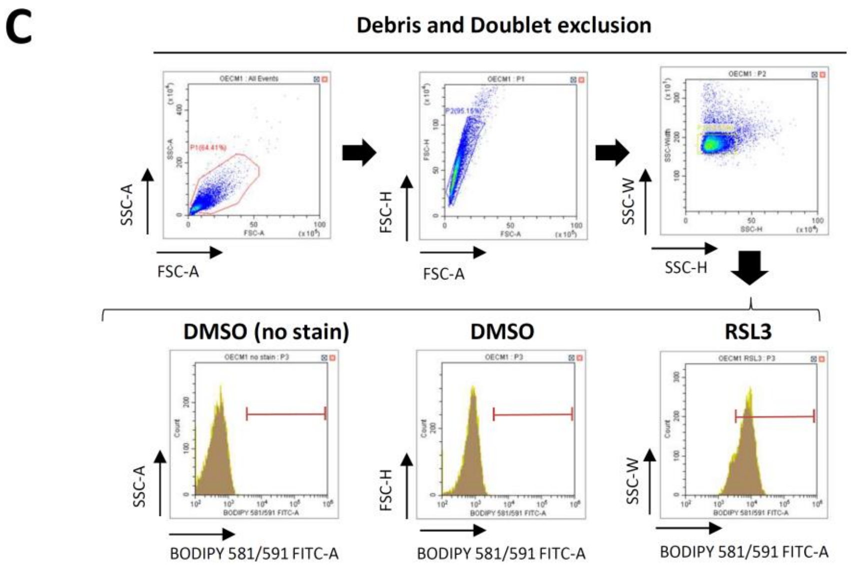

Supplementary Figure S12. Gating strategies of flow cytometry.
A, gating scheme for detection of PD-L1 expression in HSC-3 cells treated with FIN56 or DMSO control used in Fig. 3F. **B**, gating scheme for detection of infiltrated immune cells (CD4 T cell, CD8a T cell, B cell, DC, and macrophage) in FIN56 or DMSO control treated tumor cell used in Fig. 6C-D. **C**, gating scheme for detection of BODIPY 581/591 FITC lipid ROS expression in six HNSCC cell lines (FaDu, HSC-3, SAS, TE1, CAL-27, OECM-1) and a primary HNSCC culture treated with RSL3 1μM for 4h or a control vehicle DMSO. The data was used in Supplementary Fig. 4C.

Supplementary Fig. 13 Uncropped films of the experiments displayed in the figures and Supplementary figures:

Fig. 3E

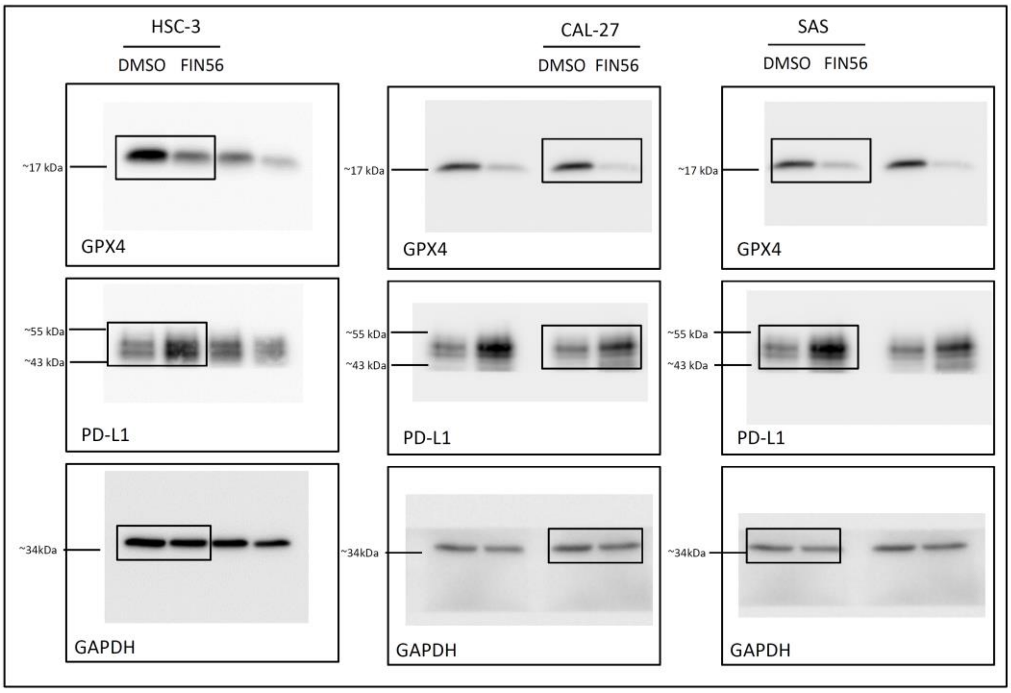

Fig. 3H

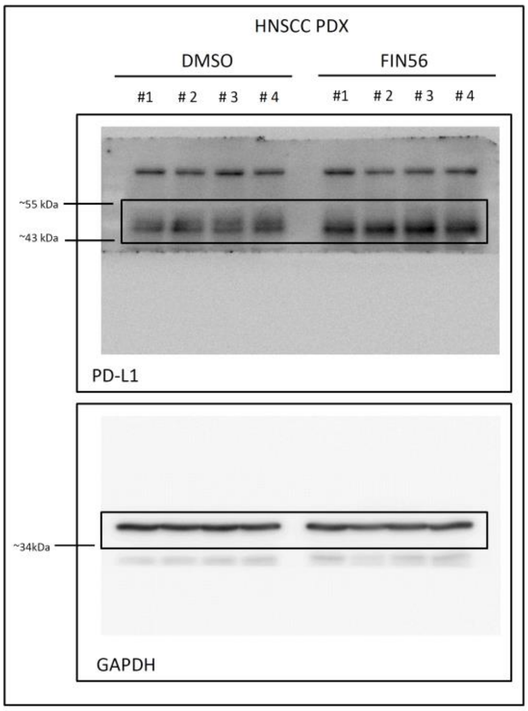

HNSCC PDX
DMSO
FIN56
#1 # 2 # 3 # 4
#1 # 2 # 3 # 4
~55 kDa
~43 kDa
PD-L1
~34kDa
GAPDH

Fig. 4B

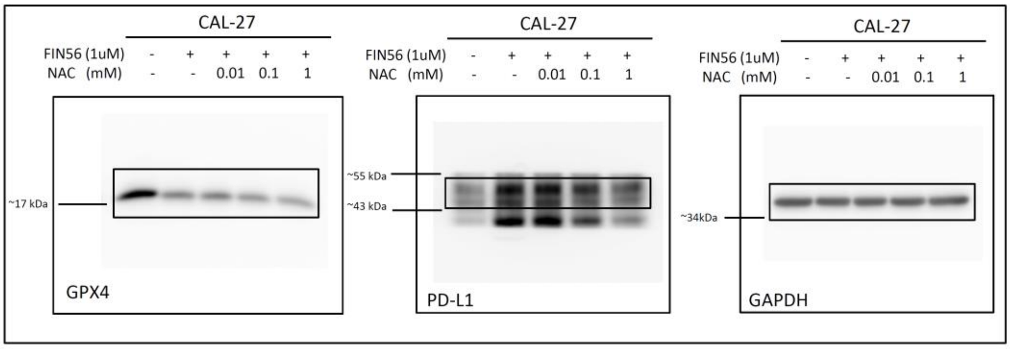

CAL-27
FIN56 (1uM) - + + + +
NAC (mM) - - 0.01 0.1 1
~17 kDa
GPX4
CAL-27
FIN56 (1uM) - + + + +
NAC (mM) - - 0.01 0.1 1
~55 kDa
~43 kDa
PD-L1
CAL-27
FIN56 (1uM) - + + + +
NAC (mM) - - 0.01 0.1 1
~34kDa
GAPDH

Fig. 4D

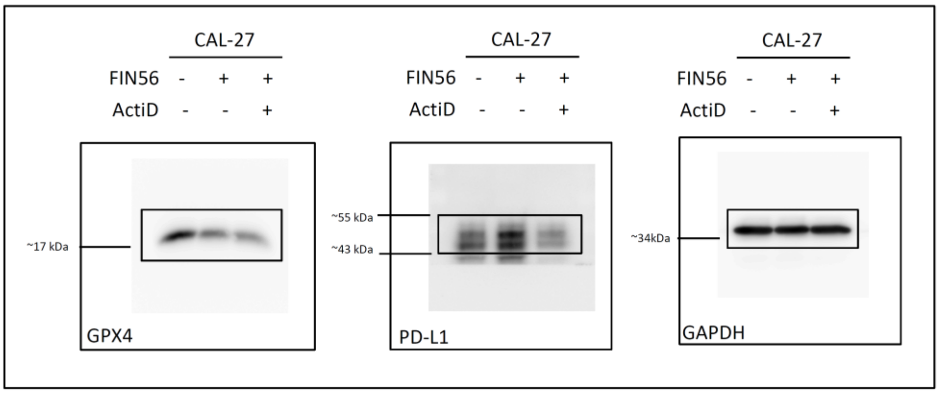
CAL-27
FIN56 - + +
ActiD - - +
~17 kDa
GPX4
CAL-27
FIN56 - + +
ActiD - - +
~55 kDa
~43 kDa
PD-L1
CAL-27
FIN56 - + +
ActiD - - +
~34kDa
GAPDH

Fig. 4E

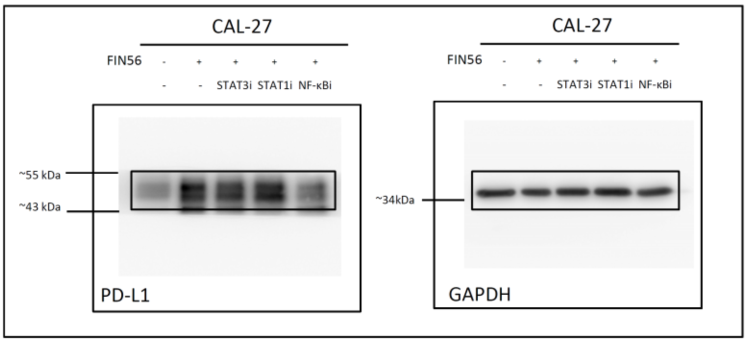
CAL-27
FIN56 - + + + +
- - STAT3i STAT1i NF-κBi
~55 kDa
~43 kDa
PD-L1
CAL-27
FIN56 - + + + +
- - STAT3i STAT1i NF-κBi
~34kDa
GAPDH

Fig. 4F

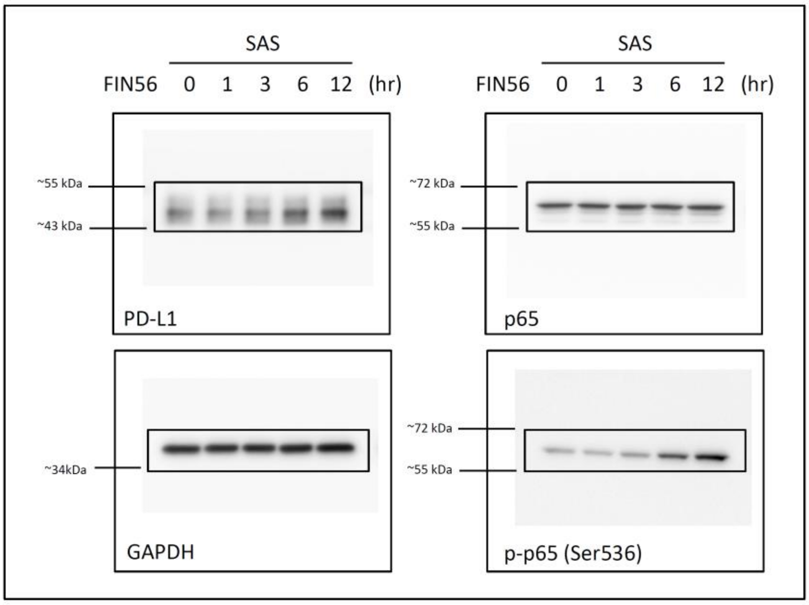

Fig. 4G

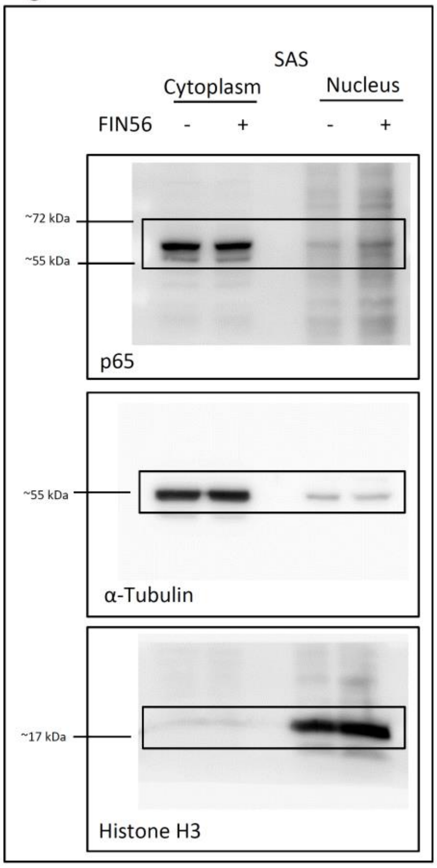

Fig. 4J

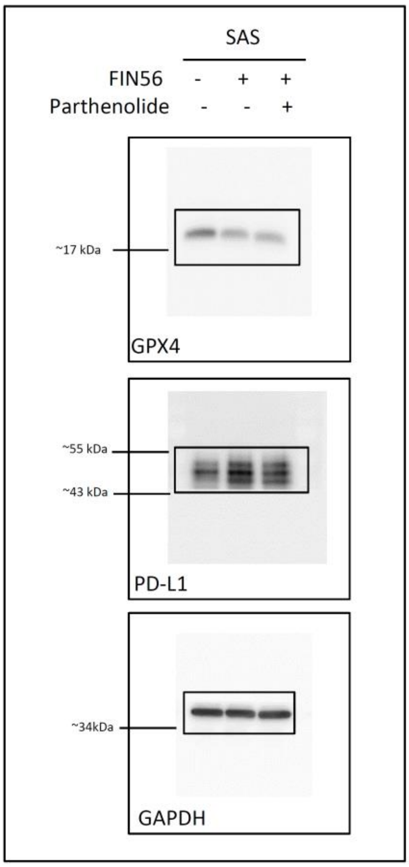

Supplementary Fig. 4B

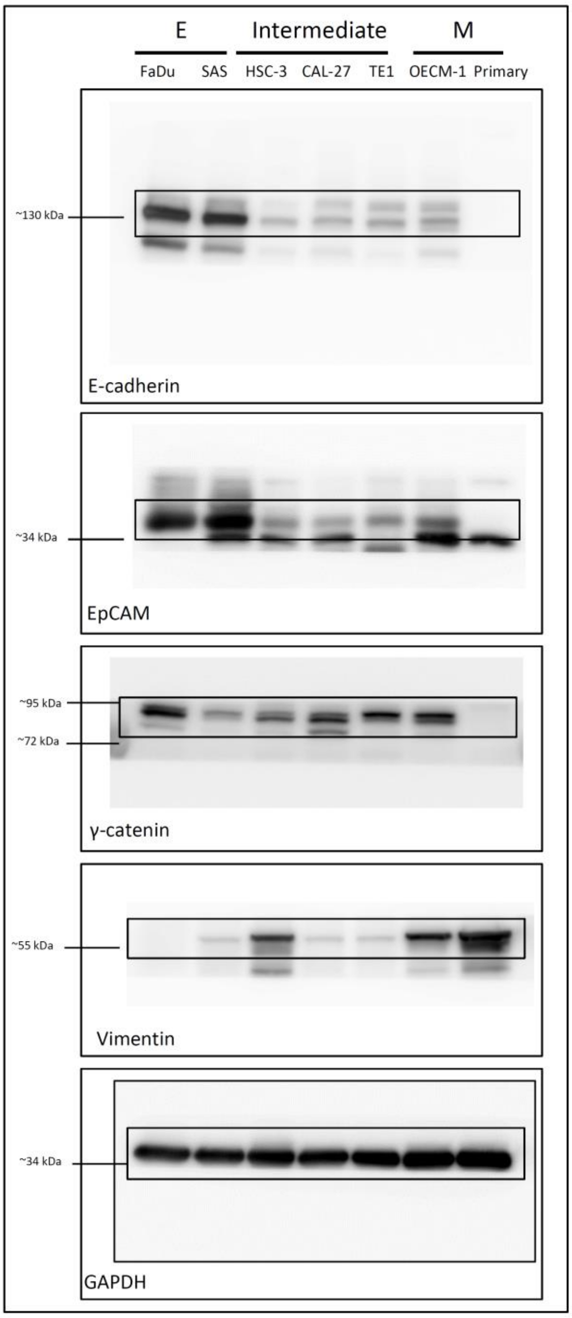

Supplementary Fig. 4F

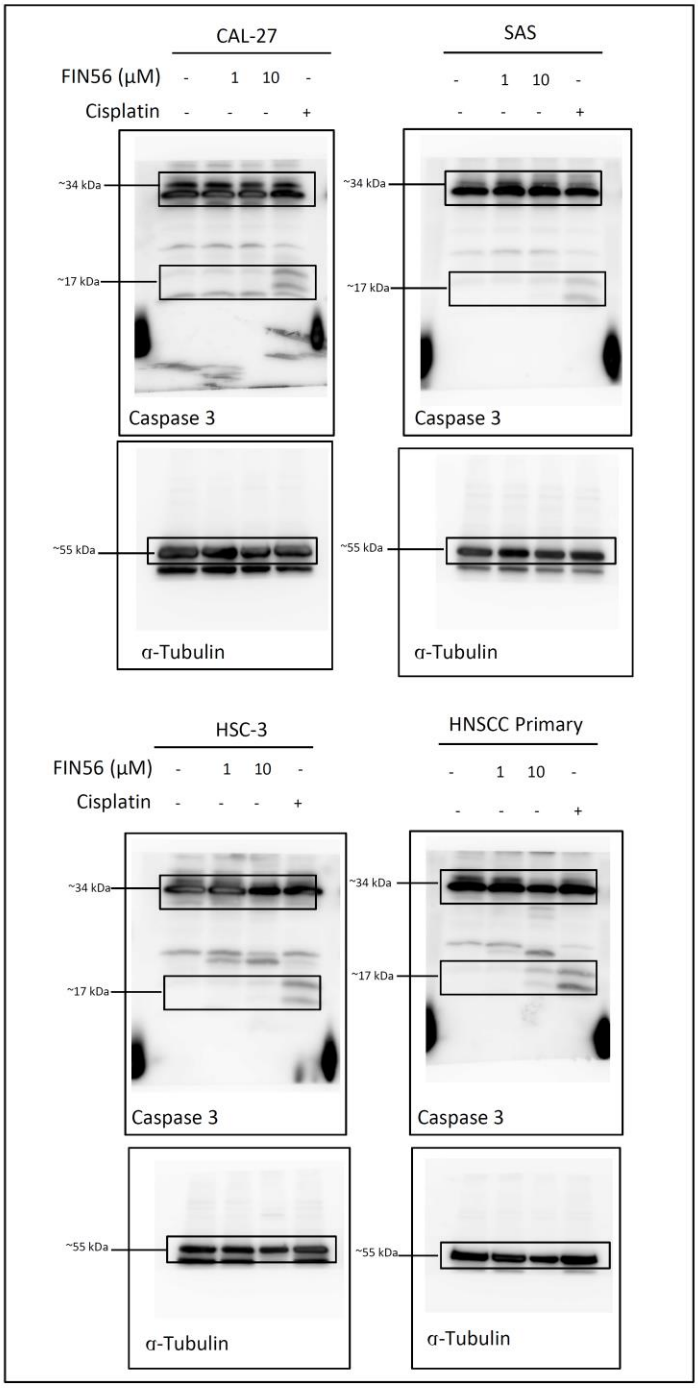

Supplementary Fig. 4F

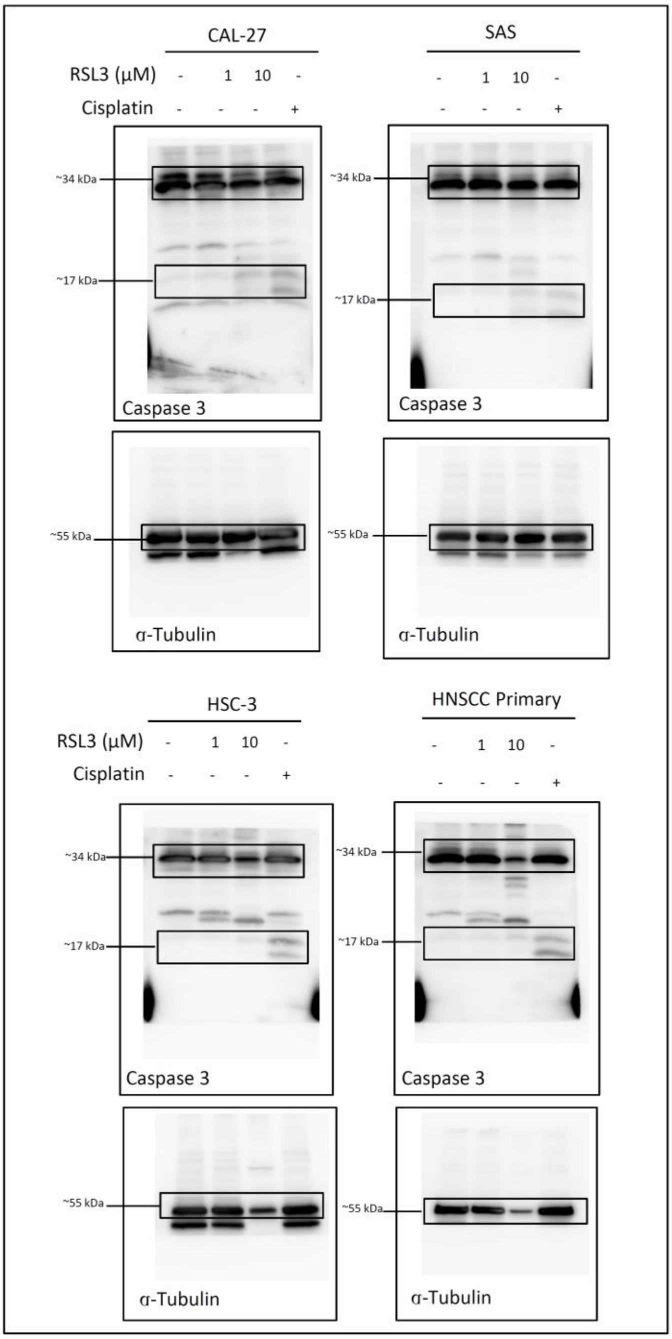

Supplementary Fig. 5B

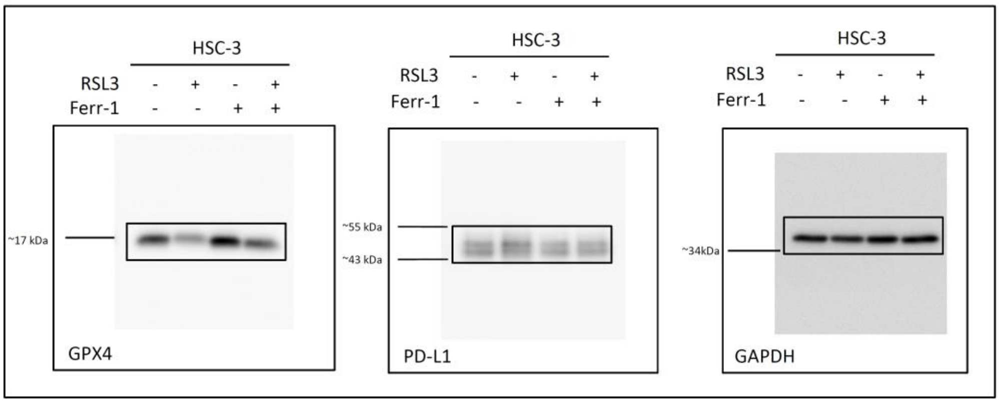

Supplementary Fig. 7C

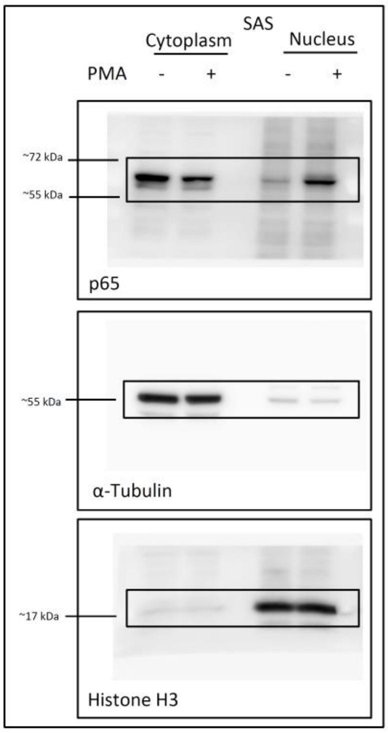

Supplementary Fig. 7D

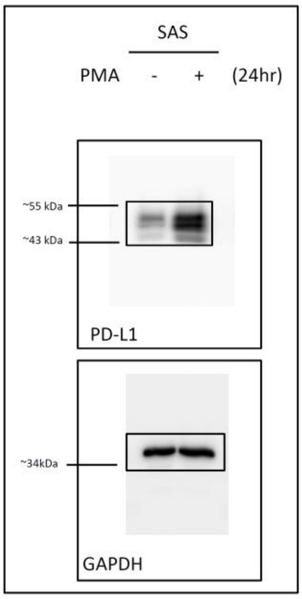

Supplementary Fig. 8H

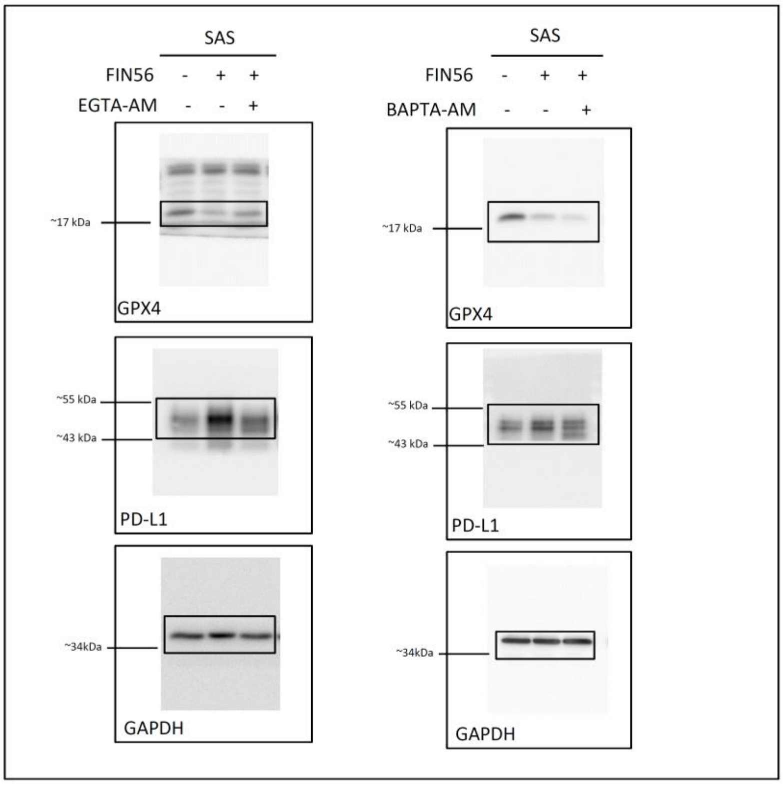

Supplementary Fig. 9B

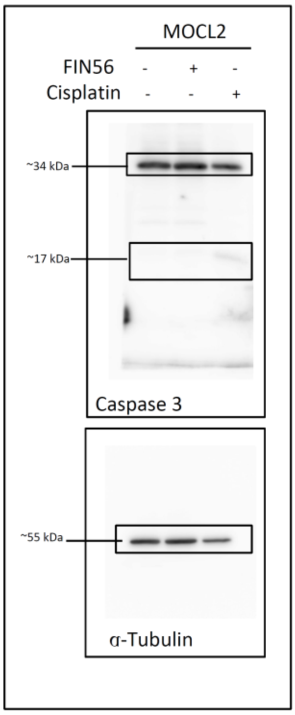

Supplementary Fig. 9C

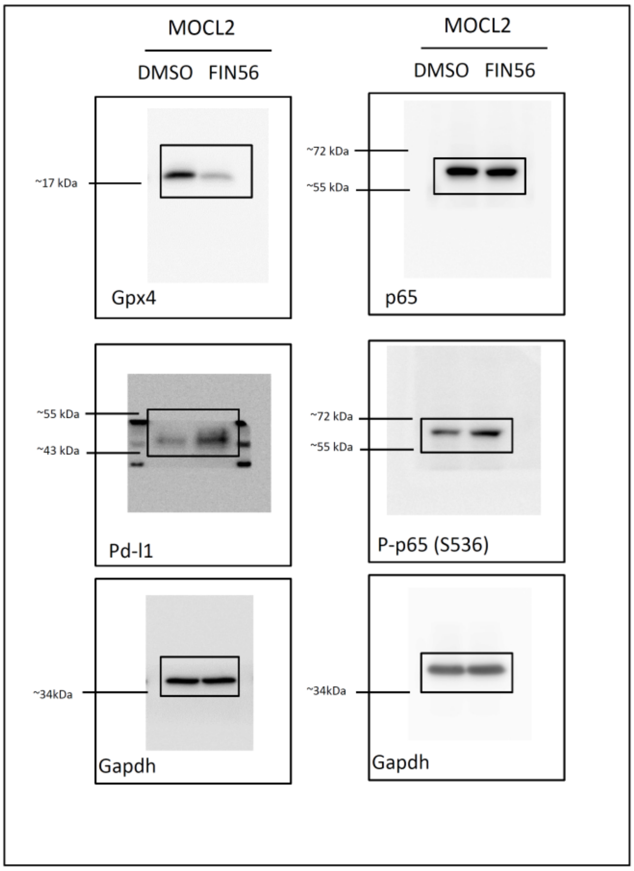

Supplementary Fig. 9D

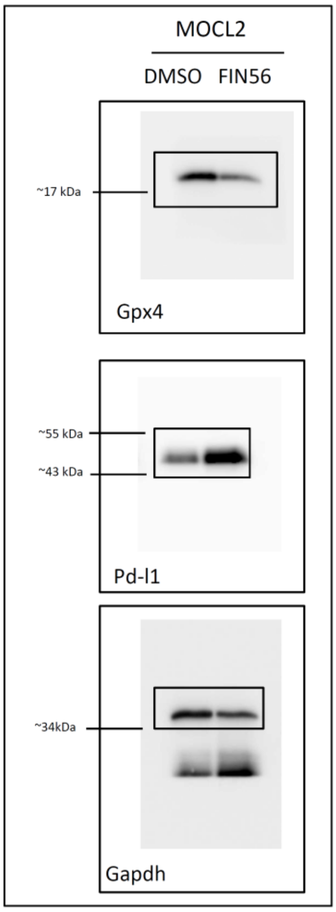

Legends for Supplementary Tables

Supplementary Table 1. Characteristics of TVGH HNSCC patients.

Supplementary Table 2. WGCNA of TVGH HNSCC for RNA sequencing.

Supplementary Table 3. Signature gene lists for enrichment analysis to annotate the gene correlation network.

Supplementary Table 4. Cell counts of subclusters of the single-cell RNA sequencing experiment.

Supplementary Table 5. Single-cell RNA sequencing of tumor cluster feature.

Supplementary Table 6. Dominantly expressed genes in HNSCC under ferroptotic stress.

Supplementary Table 7. GO analysis for ferroptosis enriched genes.

Supplementary Table 8. Information of antibodies and sequence of the oligonucleotides for RT-qPCR in this study.

Supplementary Table 9. HNSCC bulk RNA sample QC.
