# Supplementary material for: Ferroptosis Signature Shapes the Immune Profiles to Enhance the Response to Immune Checkpoint Inhibitors in Head and Neck Cancer
Source: Adv Sci (Weinh). 2023 Apr 7;10(15):2204514. doi: 10.1002/advs.202204514 (PMC10214241; doi:10.1002/advs.202204514)
Supplement: Supplementary file 9 — Supporting Information [file ADVS-10-2204514-s006.pdf]

Supplementary Table 8. Information of antibodies and sequence of the oligonucleotides for RT-qPCR in this study

**Antibodies**				
**Protein**	**Application**	**Antibody**	**Origin**	**Incorporation**
GPX4	WB, FC, IHC	ab125066	rabbit mAb	Abcam Plc. (Cambridge, UK)
GAPDH	WB	# 2118	rabbit mAb	Cell Signaling Technology, Inc. (Danvers, MA)
p65	WB	# 8242	rabbit mAb	Cell Signaling Technology, Inc. (Danvers, MA)
p65 pS536	WB	# 3033	rabbit mAb	Cell Signaling Technology, Inc. (Danvers, MA)
ɑ -Tubulin	WB	T9026	mouse mAb	Sigma-Aldrich (St. Louis, MO, USA)
PD-L1	WB, IF, VP, IHC	# 13684	rabbit mAb	Cell Signaling Technology, Inc. (Danvers, MA)
mouse PD-L1	WB	ab213480	rabbit mAb	Abcam Plc. (Cambridge, UK)
Caspase-3	WB	# 9662	rabbit pAb	Cell Signaling Technology, Inc. (Danvers, MA)
Histone H3	WB	# 9715	rabbit pAb	Cell Signaling Technology, Inc. (Danvers, MA)
PDL1-PE	FC	# 393607	mouse mAb	BioLegend, Inc. (San Diego, CA)
PE Mouse IgG1, κ Isotype Ctrl	FC	# 981804	mouse mAb	BioLegend, Inc. (San Diego, CA)
Alexa Fluor 488	IF	A27034	rabbit pAb	Thermo Fisher Scientific Inc. (Waltham, MA)
CD4	VP	MA512259	mouse mAb	Thermo Fisher Scientific Inc. (Waltham, MA)
4HNE	VP	ab46545	rabbit pAb	Cell Signaling Technology, Inc. (Danvers, MA)
CD8a	VP	MA513473	mouse mAb	Thermo Fisher Scientific Inc. (Waltham, MA)
PD1	VP	ab137132	rabbit mAb	Abcam Plc. (Cambridge, UK)
PanCK	VP	ab27988	mouse mAb	Abcam Plc. (Cambridge, UK)
CD11c	VP	ab52632	rabbit mAb	Abcam Plc. (Cambridge, UK)
CD33	VP	ab269456	rabbit mAb	Abcam Plc. (Cambridge, UK)
CD66b	VP	BD555723	mouse mAb	BD Biosciences Systems & Reagents Inc. (San Jose, CA)
CD68	VP	ab955	mouse mAb	Abcam Plc. (Cambridge, UK)
mouse PD-L1	ICB	BE0101	rat mAb	Bio X Cell (Lebanon, NH, USA)
rat IgG2b	ICB	BE0090	rat mAb	Bio X Cell (Lebanon, NH, USA)
mouse CD45-FITC	FC	FITC-65087	rat mAb	Proteintech Group, Inc. (IL, USA)
mouse CD3-APC	FC	# 100236	rat mAb	BioLegend, Inc. (San Diego, CA)
mouse CD4-PerCP	FC	# 100432	rat mAb	BioLegend, Inc. (San Diego, CA)
mouse CD8a-PE	FC	# 100708	rat mAb	BioLegend, Inc. (San Diego, CA)
mouse IFN-r-APC Cy7	FC	# 505850	rat mAb	BioLegend, Inc. (San Diego, CA)
mouse PD1-FITC	FC	# 135214	rat mAb	BioLegend, Inc. (San Diego, CA)
mouse CD11b-APC	FC	# 101212	rat mAb	BioLegend, Inc. (San Diego, CA)
mouse CD11c-BV605	FC	# 117333	rat mAb	BioLegend, Inc. (San Diego, CA)
mouse F4/80-PE	FC	# 123126	rat mAb	BioLegend, Inc. (San Diego, CA)
mouse PD-L1-BV650	FC	# 115545	rat mAb	BioLegend, Inc. (San Diego, CA)
mouse CD8a	IHC	# 98941	rabbit mAb	Cell Signaling Technology, Inc. (Danvers, MA)

mouse GZMB	IHC	# 46890	rabbit mAb	Cell Signaling Technology, Inc. (Danvers, MA)
mouse CD4	IHC	# 25229	rabbit mAb	Cell Signaling Technology, Inc. (Danvers, MA)
PD-L1	FC	# 393606	mouse mAb	BioLegend, Inc. (San Diego, CA)
CD4	FC	# 317410	mouse mAb	BioLegend, Inc. (San Diego, CA)
CD8a	FC	# 300912	mouse mAb	BioLegend, Inc. (San Diego, CA)
CD11c	FC	# 301608	mouse mAb	BioLegend, Inc. (San Diego, CA)
CD14	FC	BD557742	mouse mAb	BD Biosciences Systems & Reagents Inc. (San Jose, CA)
CD56	FC	# 318308	mouse mAb	BioLegend, Inc. (San Diego, CA)
CD19	FC	# 363030	mouse mAb	BioLegend, Inc. (San Diego, CA)

Abbreviations: FC, flow cytometry; IF, immunofluorescence; IHC, immunohistochemistry; WB, western blot; ICB, immune checkpoint blockade; VP,Vectra Polaris: mAb: monoclonal antibody; pAb, polyclonal antibody

Primers for RT-qPCR

**Gene name**		**sequence (5'-3')**	**gene name**		**sequence (5'-3')**
18S	F	CTACCACATCCAAGGAAGCA	*m18S*	F	GTAACCCGTTGAACCCCATT
	R	TTTTTCGTCACTACCTCCCCG		R	AGAGGGACGTGAGAGAGCAT
CD274	F	TCAATGCCCCATACAACAAA	*mCD274*	F	GCTCCAAAGGACTTGTACGTG
	R	TGCTTGTCCAGATGACTTCG		R	TGATCTGAAGGGCAGCATTTC
